# Overexpression of *EZH2* in multiple myeloma is associated with poor prognosis and dysregulation of cell cycle control

**DOI:** 10.1038/bcj.2017.27

**Published:** 2017-03-31

**Authors:** C Pawlyn, M D Bright, A F Buros, C K Stein, Z Walters, L I Aronson, F Mirabella, J R Jones, M F Kaiser, B A Walker, G H Jackson, P A Clarke, P L Bergsagel, P Workman, M Chesi, G J Morgan, F E Davies

**Affiliations:** 1 The Institute of Cancer Research, London, UK; 2 The Royal Marsden NHS Foundation Trust, London, UK; 3 Myeloma Institute, University of Arkansas for Medical Sciences, Little Rock, AR, USA; 4 Department of Haematology, Newcastle University, Newcastle, UK; 5 Mayo Clinic Arizona, Scottsdale, AZ, USA

## Abstract

Myeloma is heterogeneous at the molecular level with subgroups of patients characterised by features of epigenetic dysregulation. Outcomes for myeloma patients have improved over the past few decades except for molecularly defined high-risk patients who continue to do badly. Novel therapeutic approaches are, therefore, required. A growing number of epigenetic inhibitors are now available including EZH2 inhibitors that are in early-stage clinical trials for treatment of haematological and other cancers with *EZH2* mutations or in which overexpression has been correlated with poor outcomes. For the first time, we have identified and validated a robust and independent deleterious effect of high *EZH2* expression on outcomes in myeloma patients. Using two chemically distinct small-molecule inhibitors, we demonstrate a reduction in myeloma cell proliferation with EZH2 inhibition, which leads to cell cycle arrest followed by apoptosis. This is mediated via upregulation of cyclin-dependent kinase inhibitors associated with removal of the inhibitory H3K27me3 mark at their gene loci. Our results suggest that EZH2 inhibition may be a potential therapeutic strategy for the treatment of myeloma and should be investigated in clinical studies.

## Key points

1. High *EZH2* mRNA expression in myeloma patients at diagnosis is associated with poor outcomes and high-risk clinical features.
2. Specific targeting of EZH2 with well-characterised small-molecule inhibitors *in vitro* leads to upregulation of cell cycle control genes leading to cell cycle arrest and apoptosis.

## Introduction

Myeloma is a malignancy of plasma cells that accumulate in the bone marrow (BM), suppress normal haematopoiesis, lyse bone and secrete monoclonal immunoglobulin into the blood. Outcomes for many myeloma patients have improved over the past two decades with the introduction of proteasome inhibitors, immunomodulatory drugs and, more recently, monoclonal antibodies. However, high-risk disease, characterised by ⩾1 adverse cytogenetic features (t(4;14), t(14;16), t(14;20), 1q+, 17p−)^1, 2^ or distinct gene expression profiles (for example, UAMS GEP70 score)^3^ remains therapeutically intractable, with little evidence that currently available therapies have improved patient outcomes.^4^ New treatment strategies are therefore urgently required.

Myeloma is molecularly heterogeneous with a number of clear molecular subgroups defined at the DNA or gene expression level. Epigenetic modifications also have an important role in myeloma pathogenesis:^5^ one of the primary translocation events, which occurs in a high proportion of GEP70 high-risk patients, t(4;14), leads to upregulation of the histone 3 lysine 36 (H3K36) methyltransferase MMSET.^6, 7, 8, 9^ In addition, changes in DNA methylation patterns have been identified between subgroups and with advancing stages of disease.^10^

A unifying characteristic across subgroups is dysregulation of the G1/S cell cycle checkpoint mediated via overexpression of a D group cyclin.^11^ The cyclin Ds, in complex with cyclin-dependent kinase 4/6 (CDK4/6), phosphorylate Rb protein, initiating DNA transcription and driving cell proliferation. Higher rates of proliferation are associated with advanced disease stages and with high-risk compared with low-risk disease.^12, 13^ Targeting proliferation via cell cycle control proteins is, therefore, an attractive therapeutic target for such disease segments. Targeting the epigenetic events that impact on this cell cycle checkpoint could provide a novel therapeutic strategy.

EZH2 is a histone methyltransferase acting primarily at H3K27 where it catalyses the conversion to a tri-methylated mark (H3K27me3), a modification associated with the repression of gene expression.^14, 15^ The methyltransferase activity of EZH2 is specifically mediated via the SET domain of the protein.^16^ It is a member of the polycomb repressive complex (PRC2), which is comprised of EZH2 with EED, SUZ12 and RbAp48 and accessory proteins, such as JARID2 and ASXL1.^14^ The maintenance of the structure of this complex is important for the function of EZH2. The histone demethylase UTX/KDM6A, which is frequently lost in myeloma cell lines and in some patient samples,^17^ removes the H3K27me2/3 marks, counteracting the activity of EZH2.^18^

EZH2 has an important role in normal B-cell development, with the expression and H3K27me3 levels influencing differentiation decisions.^19, 20^ EZH2 expression is high in germinal centre B cells resulting in the silencing of cell cycle checkpoints and allowing B cell expansion with a subsequent reduction in EZH2, allowing cells to differentiate into plasma cells. Transformation of germinal centre cells by EZH2-activating mutations, occurring in the SET domain, has been shown to drive up to a quarter diffuse large B-cell and 10% of follicular lymphomas, circumventing normal cellular differentiation.^21^ High expression of EZH2 has also been linked to adverse outcome and aggressive tumour biology in numerous solid tumours and haematological malignancies, including breast, lung, bladder and chronic lymphocytic lymphoma.^22, 23, 24, 25, 26^ Even in diffuse large B-cell lymphoma, high EZH2 expression leads to high levels of H3K27me3, independent of the presence of a mutation and is associated with high-grade features.^27^ Inactivating mutations in the H3K27 demethylase *KDM6A* (also potentially leading to pathologically high H3K27me3) have also been identified and these, along with the presence of *ARID1A* mutations, have been suggested to sensitise cells to EZH2 inhibition.^28, 29^ Based on targeting the oncogeneic activity of EZH2, a number of specific small-molecule inhibitors have been developed with three compounds in early-phase clinical studies (http://www.clinicaltrials.gov).

We have previously analysed DNA from almost 500 cases of newly diagnosed myeloma patients and their paired germline controls.^30, 31^ No patients had mutations in *EZH2*, but 3% of patients had potentially inactivating mutations or deletions in its partner demethylase *KDM6A*, with evidence of a negative effect on patient outcomes. Previous studies have shown an increase in EZH2 expression as disease progresses from monoclonal gammopathy of undetermined significance (MGUS) through smouldering myeloma to myeloma and that genes underexpressed in multiple myeloma (MM) are associated with H3K27me3 targets in embryonic stem cells.^16, 32^ In addition, it has been suggested that MMSET overexpression in myeloma may drive genome-wide redistribution of EZH2 and H3K27me3 marks.^33^ Recent studies have indicated that myeloma cells may respond to EZH2 inhibition but with variation across cell lines and no clear biomarker predictive of response identified.^34, 35^

Using large data sets totalling almost 1500 patients in several phase III clinical trials using different therapeutic strategies, we, for the first time, identify and validate a robust and independent deleterious effect of high *EZH2* gene expression on outcomes in myeloma patients. Using two chemically distinct, specific, small-molecule inhibitors in myeloma cell lines and primary patient cells, we demonstrate EZH2 to be a therapeutic target in myeloma, including cases with high-risk features. We find that inhibition of EZH2 in myeloma induces cell cycle arrest followed by apoptosis. Analysis of mRNA and protein expression changes following inhibition suggests that this is mediated via upregulation of cell cycle control genes, the CDK inhibitors *CDKN1A*/p21 and *CDKN2B*/p15, which we demonstrate to be regulated by H3K27me3. These gene expression changes only occur at dose and time points that inhibit cellular proliferation, suggesting that they could be used as a biomarker of efficacy. Our study demonstrates that EZH2 inhibition may be an effective therapeutic strategy for myeloma patients, potentially even for those with high-risk disease for whom current approaches are ineffective.

## Materials and Methods

### Expression and survival analysis

Gene expression profiling (GEP) was performed for patients enrolled in the MRC Myeloma IX trial (MyIX, *n*=259, GSE21349) and Total Therapy trials (UAMS-TT, *n*=1230, GSE2658, GSE38627) as previously described.^1, 13, 36, 37, 38, 39, 40, 41, 42^ These trials used very different induction treatment regimens (Supplementary Methods). Expression of EZH2 was measured using the 203358_s_at probeset, confirmed to have good coverage, sensitivity and specificity for EZH2 (UCSC genome browser, www.genome.ucsc.edu and GeneAnnot from Genecard, ww.genecards.weizmann.ac.il/geneannot). The optimal cut point was defined for EZH2 in each data set, and Kaplan–Meier curves were drawn to compare high expressers to low expressers. Survival was compared using the Logrank statistic. Univariate and multivariate analysis with Cox proportional hazards regression was performed within each data set. All covariates considered in the univariate model were included in the multivariate model. Analysis was carried out using R 3.2.3,^43^ SAS 9.4 (SAS Institute, Cary, NC, USA) and SPSS 22 (IBM, New York, NY, USA). A probability value of <0.05 was considered statistically significant. Expression was compared across the UAMS subgroups^13^ and between GEP70 low- and high-risk groups^3^ in the Total Therapy trials. Correlation between EZH2 expression and both the GEP70 absolute score and proliferation index^12, 13^ was also calculated.

### Cell lines and reagents

MM cell lines, KMS11, JIM3, MM1.S, LP1, KMS12BM, RPMI8226, KMM1 and U266, were grown in RPMI1640 medium containing GlutaMax (Gibco, Thermo Fisher Scientific, Waltham, MA, USA) supplemented with 10% heat-inactivated fetal bovine serum (Gibco, Thermo Fisher Scientific). The HS5 stromal cell line was grown in Dulbecco's modified Eagle's medium (Gibco, Thermo Fisher Scientific) supplemented with 10% heat-inactivated foetal bovine serum. This cell line was transfected with a green fluorescent protein (GFP) short hairpin RNA viral vector to produce the stable HS5-GFP cell line. GFP expression was confirmed to be >95% prior to each experiment. Cell line identity was confirmed using single tandem repeat analysis and cells tested negative for mycoplasma (PCR Mycoplasma Test Kit, Promokine, Heidelburg, Germany). Cell cultures were kept at 37 °C in a humidified gas chamber with 95 air and 5% carbon dioxide.

UNC1999 and UNC2400 were a kind gift from The Structural Genomics Consortium, Toronto, ON, Canada. EPZ005687 was synthesised in house.^44^ Compounds were dissolved in dimethyl sulphoxide (DMSO) to a 50 mM stock solution and stored in aliquots at −20 °C.

### Primary patient samples

BM aspirate samples were obtained from relapsing myeloma patients with consent after approval by The Royal Marsden review board. The white cell layer was separated by density gradient centrifugation using Ficoll-Paque Premium (GE Healthcare UK Ltd, Little Chalfont, Buckinghamshire, UK). CD138+ plasma cells were selected using antibodies conjugated to magnetic beads (Miltenyi-Biotech, Bergish Gladbach, Germany). Purity was confirmed to be >90% by microscopy following cytospin and modified Wrights staining (Haematek, Siemens Healthcare, Erlangan, Germany). Patient sample molecular features were characterised using quantitative reverse transcriptase-PCR (qRT-PCR) and multiplex ligation-dependent probe amplification as previously described.^45, 46^ Peripheral blood mononuclear cells were separated from whole blood using Ficoll-Paque Premium (GE Healthcare UK Ltd, Little Chalfont, Buckinghamshire, UK) by density gradient centrifugation.

### Cell viability, cell cycle and apoptosis assays

Inhibition of proliferation in cell lines was measured using the *in vitro* WST-1 colorimetric assay (Roche, Penzberg, Germany). Propidium iodide (PI) staining followed by flow cytometry (LSRII flow cytometer, Beckton Dickinson, Oxford, UK) was used to analyse the cell cycle. Annexin V/PI staining was used to look for evidence of apoptosis with the proportion of Annexin V plus Annexin V- and PI-positive cells (compared with DMSO control) calculated. Flow cytometric data was analysed using the BD FACSDiva software (Beckton Dickinson, Franklin Lakes, NJ, USA) and/or FlowJo (Ashland, OR, USA). The Caspase-Glo 3/7 luminescent assay (Promega, Madison, WI, USA) was used to compare caspase activity after cell number was normalised.

### Co-culture of BM stroma and CD138+ patient plasma cells

HS5-GFP and CD138+ cells were co-cultured in a 1:5 ratio and incubated for 72 h with the indicated compound/control. Flow cytometric analysis was performed as above but with Annexin V and 4',6-diamidino-2-phenylindole (DAPI) instead of PI (to avoid crossover with GFP signal) and by gating on only GFP-negative cells to assess Annexin V/DAPI staining in the myeloma cells. Viability was determined by the proportion of Annexin V- and DAPI-negative cells (compared with DMSO control).

### Chromatin immunoprecipitation–PCR (ChIP-PCR)

ChIP-PCR was performed using the ChIP-IT Express Enzymatic Kit (Active Motif, Carlsbad, CA, USA) with optimisations/modifications as described in Supplementary Methods.

Primers were designed using Primer Express (Thermo Fisher Scientific, Waltham, MA, USA) at the transcriptional start site (TSS) and the promoter (PROM) region of each gene of interest at areas that appeared marked with regulatory/PROM elements using tracks from ENCODE on UCSC genome browser. Additional primers were designed to regions approximately 5 Kb upstream of the TSS and not marked by ENCODE tracks. Positive and negative control primer sets were also run (Active Motif). Percentage of input calculations for each sample were made using the standard curve and the comparative C_T_ method. All samples were run in duplicate and the mean taken for each experiment. ChIP-PCR was repeated with a second complete biological replicate.

Methods for western blotting and qRT-PCR are given in Supplementary Methods.

## Results

### High *EZH2* mRNA expression is associated with worse outcomes for patients and molecular features of high-risk disease

**Figure 1:**
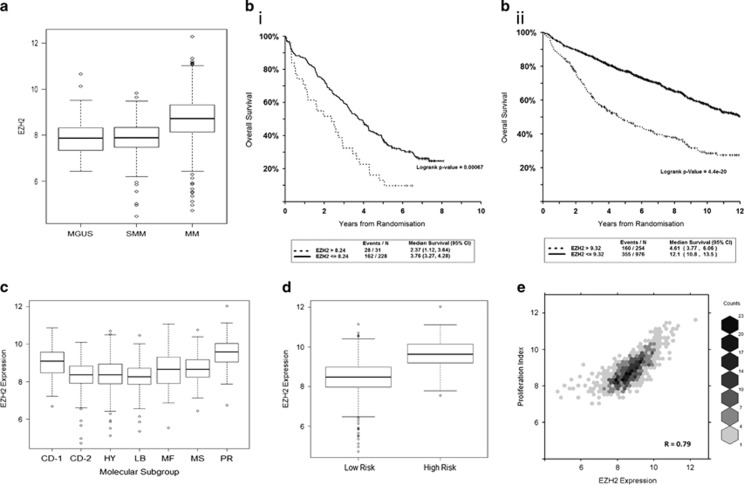
High expression of *EZH2* is associated with poor patient outcomes and features of high-risk and proliferative disease. (**a**) Box and whisker plot showing *EZH2* expression in UAMS data set (MGUS *n*=114, SMM *n*=163, MM *n*=1344) Log 2 expression values. One-way analysis of variance (F ratio 84.53, *P*<2e-16) followed by Tukey's multiple comparison test demonstrated significant difference between MGUS and MM (adj. *P*⩽0.00001) and between SMM and MM (adj. *P*⩽0.00001). (**b**) Kaplan–Meier curves showing OS (i) in the MyIX data set comparing high *EZH2* mRNA (>8.24, log 2 expression value, *n*=31) to all others (*n*=228). Median OS 2.37 years (95% CI (1.12, 3.64)) vs 3.76 (95% CI (3.27, 4.28)). Logrank *P*=0.00067 and (ii) in the UAMS-TT data set comparing high *EZH2* (>9.32, log 2 expression value *n*=254) to all others (*n*=967). Median OS 4.61 years (95% CI (3.77, 6.06)) vs 12.1 (95% CI (10.8, 13.5)). Logrank *P*=4.4e-20. (**c**) Box and whisker plot showing the *EZH2* expression across the UAMS molecular subgroups. Log 2 expression values. PR mean expression 9.56 vs all other 8.48, one-sided Welch's *t*-test *P*=5.953e-42. (**d**) Box and whisker plots showing the *EZH2* expression across UAMS GEP70 high- vs low-risk patients. High-risk GEP70 had a higher mean expression of *EZH2* (9.61 vs 8.46, one-sided Welch's *t*-test *P*-value=1.993e-40). GEP70 scores also significantly correlated with *EZH2* expression (Pearson correlation of 0.611). (**e**) High-density scatter plot demonstrating the correlation between the *EZH2* expression and the gene expression-defined proliferation index. *R*=0.79, *P*<0.0001.

Given the evidence associating EZH2 expression and oncogenesis in other cancers, we investigated its effect in myeloma patients. Using a much larger data set than previously reported (UAMS *n*=1621), we confirmed an increase in the expression of *EZH2* mRNA as myeloma progresses with the highest expression in symptomatic myeloma (MGUS vs MM adjusted *P*<0.00001, smouldering multiple myeloma (SMM) vs MM adjusted *P*<0.00001, MGUS vs SMM not significant; Figure 1a). We therefore went on to assess the effect of *EZH2* expression on clinical outcomes. In two large independent data sets of phase III clinical trial patients, MyIX *n*=259 and UAMS-TT *n*=1230, we found that high expression of *EZH2* was associated with shortened progression-free and overall survival (Figure 1b and Supplementary Figure S1), with a reduction in median overall survival from 3.76 to 2.37 years (Logrank *P*=0.00067) and from 12.1 to 4.61 years (Logrank *P*=4.4e-20), respectively. In these studies, patients received very different treatment regimens, suggesting that this effect is independent of therapy and persists even with immunomodulatory drug/proteasome inhibitor combination treatment used in the Total Therapy studies. In both data sets, the impact of *EZH2* expression remained significant on multivariate analysis (Supplementary Tables S1 and S2), demonstrating its effect is independent of other factors known to affect survival in myeloma patients including International Staging System, GEP70 risk and adverse cytogenetics.

*EZH2* expression was significantly higher in the UAMS molecularly defined PR subgroup,^13^ which is characterised by the overexpression of cancer-testis antigens, cell cycle and proliferation-related genes (Figure 1c), and was significantly higher in GEP70 high-risk patients (Figure 1d). There was also a marked correlation with a gene expression-defined proliferation index (Figure 1e, *R*=0.79, *P*<0.0001).^12^ This suggests *EZH2* expression could contribute to the high-risk phenotype. We therefore sought to investigate the effect of EZH2 inhibition in myeloma *in vitro*.

In order to examine the effect of EZH2 inhibition with EZH2 *in situ* in the PRC complex, we used the well-characterised small-molecule inhibitors available, rather than taking a knockdown approach that would disrupt the whole complex. In this study, we utilised two chemically distinct EZH2 inhibitors, EPZ005687^44^ and UNC1999,^47, 48^ along with a paired inactive analogue, UNC2400, in order to robustly demonstrate the effects to be EZH2 specific.

### EZH2 inhibition reduces myeloma cell viability in a time- and concentration-dependent manner

**Figure 2:**
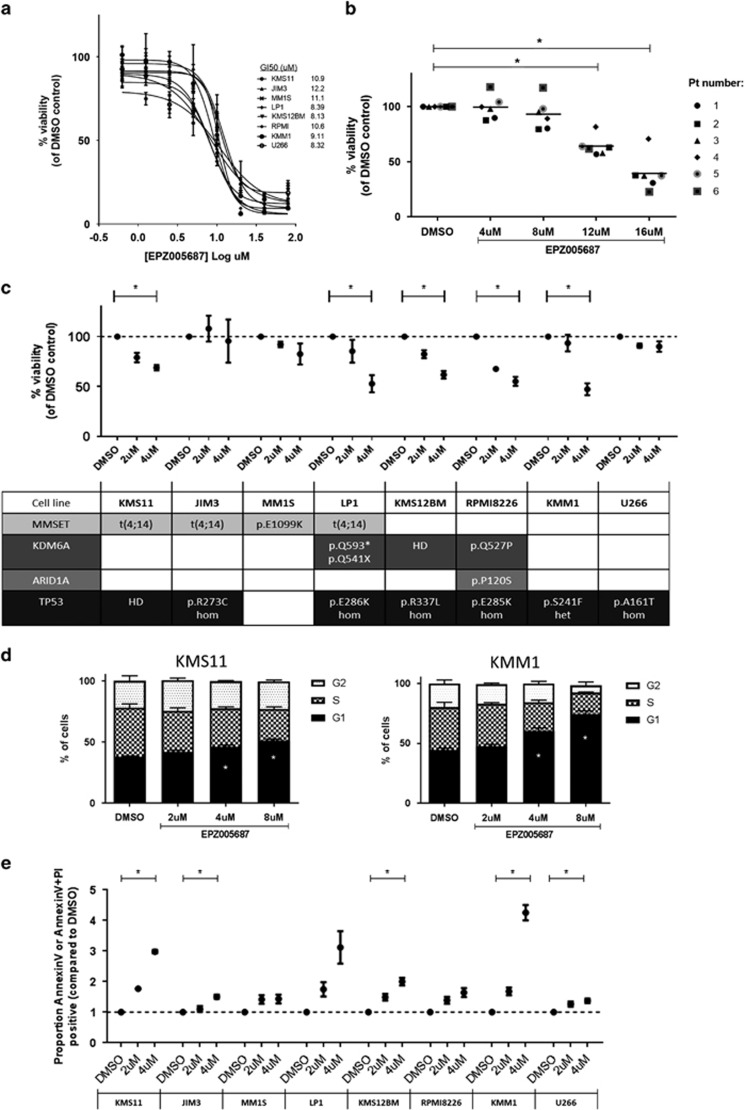
Specific EZH2 inhibition is efficacious *in vitro* in both myeloma cell line and primary patient samples, even those with high-risk features. EZH2 inhibition induces cell cycle arrest followed by apoptosis. (**a**) Cell viability determined using the WST1 assay (normalised to DMSO control) in a panel of eight myeloma cell lines incubated with increasing concentrations of EZH2 inhibitor (EPZ005687) for 72 h. The GI50 for each cell line (calculated using the Graphpad Prism software) is shown. There was no statistically significant difference between the GI50s for each cell line (one-way analysis of variance (ANOVA) *P*>0.05). Graph shows mean and s.e.m. of at least three independent biological replicates. (**b**) CD138 selected plasma cells from six patients' BM aspirate samples were co-cultured with the BM stromal cell line HS5 (GFP tagged) for 72 h in the presence of the indicated concentration of EPZ005687 or vehicle control (DMSO). Cells were then stained with Annexin V and DAPI prior to flow cytometric analysis. Results show the percentage of cell viability (of DMSO) measured as the percentage of cells that were Annexin V and PI-negative within the GFP-negative fraction. Raw data and mean value (horizontal line) are shown. One sample *t*-tests were performed to look for a significant reduction in viability at each concentration compared with 100%. Those with *P*-values<0.05 are indicated by an asterisk (*). (**c**) Cell viability determined using the WST1 assay (normalised to DMSO control) in a panel of eight myeloma cell lines incubated with increasing concentrations of EZH2 inhibitor (EPZ005687) for 6 days. Graph shows mean and s.e.m. of at least three independent replicates in each cell line. The cell line features of factors previously demonstrated to be relevant to EZH2 inhibition in myeloma and TP53 status are shown in the table below with further details in Supplementary Table S3. Of note, no cell lines used had *EZH2* mutations (details from Broad CCLE, MMRF Myeloma Cell Line Characterization Data repository and van Haaften *et al.*^17^). HD=homozygous deletion, hom=homozygous mutation, het=heterozygous mutation. One sample *t*-tests were performed to look for a significant reduction in viability at 4 μM compared with 100%. Those with *P*-values<0.05 are indicated by an asterisk (*). (**d**) Cell cycle analysis with propidium iodide staining was performed following EZH2 inhibition with EPZ005687 for 3 days in KMS11 and KMM1 cell lines. The cells in each phase of the cell cycle are shown as a percentage of all cells in cycle. Results shown mean and s.e.m. of three independent replicate experiments. The mean percentage of cells in G1 was compared across conditions using a one-way ANOVA followed by multiple comparisons to DMSO control. There was a significant increase (adj. *P*<0.05) in G1% indicated by an asterisk (*). (**e**) Apoptosis was assessed after 6 days of incubation with increasing concentrations of EPZ005687 and compared with DMSO control by Annexin V/PI staining in the panel of eight cell lines. One sample *t*-tests were performed to look for a significant increase at each concentration compared with 1. Statistical significance (*P*<0.05) is indicated by an asterisk (*).

We first investigated the effect of EZH2 inhibition with EPZ005687 over 72 h using the colourimetric WST-1 viability assay in a panel of eight cell lines all of which were confirmed to express EZH2 by immunoblotting (Supplementary Figure S2A). EPZ005687 reduced viability in all myeloma cell lines in a time- and concentration-dependent manner even in those with high-risk features, such as t(4;14)/t(14;16), *TP53* mut/del or both. (Figure 2a, Supplementary Figure S2B and Supplementary Table S3). The GI50s were similar across cell lines (one-way analysis of variance *P*>0.05) at 72 h (8–12 uM).

In order to assess the effect of EZH2 inhibition in a more physiological setting, we co-cultured primary patient samples with BM stromal cells to simulate the protective BM microenvironment. We assessed viability using flow cytometry after 72 h of co-culture in the presence of EPZ005687 and found similar responses in patient cells to those seen in cell lines. (Figure 2b and Supplementary Figure S2C). These responses were seen despite heavy pretreatment of patients (median two prior lines of therapy, Table 1), and several samples carried at least one high-risk molecular feature, such as 17p−, 1q+ or t(4;14). We demonstrated a smaller reduction in viability at similar concentrations of EPZ005687 in normal donor peripheral blood mononuclear cells and BM stromal cells cultured alone (Supplementary Figures S2E and F). This suggests that a therapeutic index exists for the response to EZH2 inhibition between myeloma and non-malignant cells.

Given the previously published work looking at EZH2 inhibition in lymphoma over longer time periods, we extended our viability assays to 6 days using EPZ005687 at lower concentrations.^44, 49^ We demonstrated a reduction in viability following EZH2 inhibition, at this time point, in most cell lines using the WST-1 viability assay (Figure 2c) with no evidence of response in only 2/8 cell lines (JIM3 and U266). Our studies did not reveal any of the previously reported dependencies of EZH2 inhibition on the presence of high levels of MMSET,^33^ a *KDM6A* mutation or deletion^26^ or an *ARID1A* mutation.^29^ Neither was response related to the cell doubling time or level of baseline EZH2 or H3K27me3 expression (Supplementary Figures S2A and D, Supplementary Table S4).

Next, in order to confirm that our results were due to specific inhibition of EZH2 we repeated our viability experiments using the chemically distinct inhibitor UNC1999 and its negative control compound UNC2400. At both 3 and 6 days, we demonstrated inhibition of proliferation with UNC1999 at slightly lower concentrations than EPZ005687 (Supplementary Figures S3A and C). A lack of response with the negative control compound UNC2400 and the same pattern of response across cell lines, with JIM3 cells not responding at 6 days, confirmed that this effect was most likely due to specific inhibition of EZH2 methyltransferase activity (Supplementary Figures S3B–D).

We selected the KMS11 and KMM1 cell lines to study in further detail as representative responsive cell lines at 6 days, one from the t(4;14) subgroup and one with none of the features previously suggested to confer sensitivity to EZH2 inhibition.^28, 29, 33^

### EZH2 inhibition mediates its antiproliferative effect by inducing cell cycle arrest followed by apoptosis

We next sought to identify the mechanism by which EZH2 inhibition exerts its antiproliferative effect. Using cell fixation followed by PI staining, we demonstrated cell cycle arrest at the G1 phase following 3 days of EZH2 inhibition (Figure 2d). At 6 days, we found evidence of apoptosis by flow cytometry with an increase in Annexin and Annexin V/PI staining with increasing concentrations of EPZ005687 (Figure 2e). We confirmed this finding by demonstrating an increase in cells in <G1 on cell cycle analysis (Supplementary Figure S4A), an increase in caspase activity using the luminescent CaspaseGlo 3/7 assay (Supplementary Figure S4B) and poly ADP-ribose polymerase cleavage by immunoblotting (Supplementary Figure S4C).

### EZH2 inhibition upregulates cell cycle control genes to exert its antiproliferative effect by removing the inhibitory H3K27me3 mark

**Figure 3:**
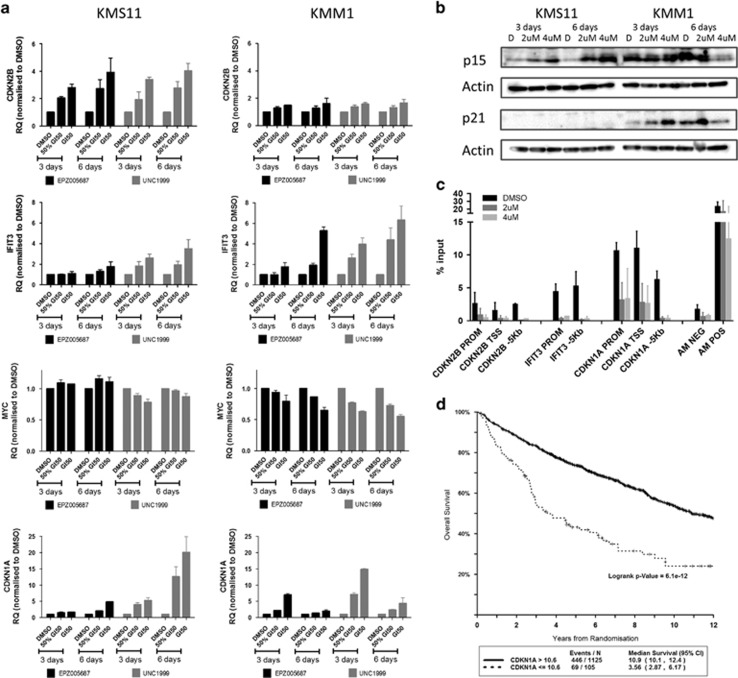
EZH2 inhibition upregulates cell cycle control genes to exert its antiproliferative effect. (**a**) Fold change in mRNA levels in EPZ005687- and UNC1999-treated KMS11 and KMM1 cell lines at 3 and 6 days, compared with DMSO control at the same time point, measured by qRT-PCR. Graphs show mean and s.e.m. for at least three independent replicate experiments. GAPDH was used as the internal control. (**b**) p15 and p21 immunoblotting of lysates from KMS11 and KMM1 cell lines after 3 and 6 days' incubation with EPZ005687. Actin was used as the loading control. Blots shown are representative of two independent experiments. (**c**) ChIP with H3K27me3 antibody followed by qRT-PCR at regions indicated in samples incubated for 6 days with DMSO control or EPZ005687 at the indicated concentrations. Pulldown of genes is shown as the percentage of input. Active motif negative and positive controls were used (mapping to genes ACTB and MYT1, respectively). An additional region approximately 5 kb (see supplementary Methods) upstream of each gene of interest was also assayed. Isotype control antibody led to negligible pulldown (data not shown). (**d**) Kaplan–Meier curves showing OS in the UAMS-TT data set comparing patients with low *CDKN1A* expression (<10.6, log 2 expression value *n*=105) to all others (*n*=1125). Median OS 3.56 years (95% CI (2.87, 6.17)) vs 10.9 (95% CI (10.1, 12.4)). Logrank *P*=6.1e-12.

Halting proliferative drive, allowing cells to exit the cell cycle, is necessary for cell differentiation and/or apoptosis. In addition to our finding of cell cycle arrest following EZH2 inhibition, Affymetrix gene expression arrays (U133plus2.0) in KMS11 and KMM1 cell lines demonstrated upregulation of genes relating to cell cycle control following treatment with EPZ005687 (Supplementary Tables S5 and S6). In the KMS11 cell line, one of the most significantly upregulated genes was *CDKN2B*, a CDK inhibitor known to inhibit cyclin D/CDK complexes in G1.^50^ In KMM1, *IFIT3* had increased expression following EZH2i. *IFIT3* has previously been demonstrated to upregulate *CDKN1A* via downregulation of *MYC* and directly upregulate *CDKN1B*.^51^ We validated these findings by incubating cells over 3 and 6 days with EPZ005687 or UNC1999 and then analysing gene expression changes using qRT-PCR. In KMS11, there was evidence of upregulation of both *CDKN2B* and the *IFIT3/MYC/CDKN1A* pathway. In KMM1, only the *IFIT3/MYC/CDKN1A* pathway appeared to be upregulated (Figure 3a). Using the negative control compound UNC2400, we confirmed that these findings were EZH2 specific (Supplementary Figure S5). mRNA changes were also demonstrated at the protein level by immunoblotting (p15 for *CDKN2B* and p21 for *CDKN1A*; Figure 3b).

To further determine whether the gene expression changes were mediated by a change in the H3K27me3 status of the gene, we performed ChIP-PCR using a validated antibody for H3K27me3 (Active Motif, no. 61017) and isotype control. Following incubation of KMS11 cells with EPZ005687 over 6 days, we identified changes in H3K27me3 in the PROM and TSS regions of *CDKN2B*, *IFIT3* and *CDKN1A* (Figure 3c). The most specific changes, however, occurred at the *CDKN1A* PROM/TSS, which were more heavily marked with H3K27me3 at baseline compared with a region approximately 5 Kb upstream. Pulldown in the isotype control-incubated samples was negligible.

These results suggest that *CDKN1A* expression might be controlled by changes in H3K27me3 and so we explored the effect of *CDKN1A* mRNA expression in our patient data sets. We found the expression of *EZH2* and *CDKN1A* to be inversely correlated (*R*=−0.170, *P*<0.0001, Supplementary Figure S6), supporting the hypothesis that *CDKN1A* expression is suppressed by increased H3K27me3 as a result of high expression of *EZH2*. In addition, low expression of *CDKN1A* was associated with a significantly shorter progression-free and overall survival (Figure 3d).

### Changes in the expression of cell cycle control genes correlate with the antiproliferative effect of EZH2 inhibition

**Figure 4:**
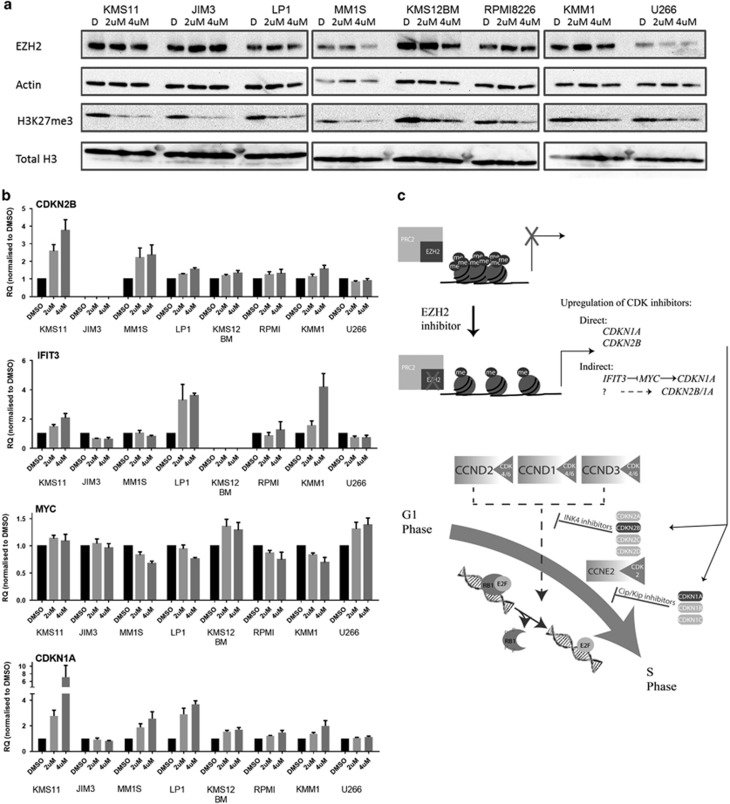
Global changes in H3K27me3 are associated with cell context-specific gene expression changes. (**a**) Immunoblotting of EZH2 and H3K27me3 from whole-cell lysates following 6 days' incubation with EPZ005687 across the panel of eight cell lines. Actin and total H3 were used as the loading controls, respectively. (**b**) Fold change in mRNA levels measured by qRT-PCR in EPZ005687-treated cell lines at 6 days, compared with DMSO control. Graphs show mean and s.e.m. for at least three independent replicate experiments. GAPDH was used as the internal control. *CDKN2B* in JIM3 cells and *IFIT3* in KMS12BM cells were not expressed at levels to allow reliable quantification of any change in the expression. Additional gene expression results are shown in Supplementary Figure S8. (**c**) Putative mechanism of action of EZH2 in myeloma cell lines. The upper part of the diagram demonstrates the change in chromatin structure in the presence of active vs inhibited EZH2, resulting in a change in the methylation status of H3K27. Where EZH2 is not inhibited, H3K27me3 is high and chromatin structure is closed preventing gene transcription. EZH2 inhibition removes methyl marks, chromatin relaxes and genes affected by the H3K27me3 mark are able to be transcribed. We show that *CDKN1A* and *CDKN2B* may be directly under the control of H3K27me3 or their transcription might be altered as a downstream result of the expression of another gene being altered. The lower part of the diagram demonstrates the cyclin D/CDK and cyclin E/CDK complexes driving cell proliferation in myeloma at the G1/S checkpoint. Upregulation of the CDK inhibitors can inhibit these complexes preventing passage of cells from G1 to S phase as shown.

In order to identify a potential biomarker of response, we next looked across our original panel of eight myeloma cell lines incubating cells over 6 days, a time point at which we had seen the most variability in response to EZH2 inhibition. Using whole-cell lysates, we first examined global changes in EZH2 and H3K27 methyl marks (Figure 4a and Supplementary Figure S7A). No consistent change in EZH2 expression was seen following inhibition; however, a marked reduction in H3K27me3 occurred across all cell lines. Changes in H3K27me2 mirrored H3K27me3, whereas in contrast H3K27me1 does not appear to be altered (Supplementary Figure S7B), supporting previous findings in other tumour types.^52^ These studies confirmed the modulation of the expected target activity of the inhibitor across cell lines but did not differentiate between responses—suggesting that global changes in methyl marks cannot be used as a predictive biomarker. Instead differential response must be due to varied effects of H3K27me3 alteration dependent on different transcriptional networks determined by the cellular context.

We, therefore, examined the transcriptional profiles across all eight cell lines in response to EZH2 inhibition at 6 days using qRT-PCR for the genes examined above plus other genes involved in cell cycle control (Figure 4b and Supplementary Figure S8). We identified a consistent increase in the expression of the CDK inhibitor *CDKN1A* in responding cell lines. This increase was independent of the *TP53* status of the cell line but was not consistently associated with an increase in *IFIT3* and decrease in *MYC* expression that had been seen in the KMM1 and KMS11 cell lines—indicating involvement of the *IFIT3/MYC/CDKN1A* pathway. This suggests that *CDKN1A* might be directly regulated by H3K27me3 changes (as is suggested by our ChIP-PCR results in KMS11) as well as indirectly via *IFIT3/MYC*, with variation between cell lines. In some cell lines (KMS11, MM1S, LP1 and KMM1), there was also an increase in the expression of *CDKN2B*. There was no evidence of a direct action of *IFIT3* upregulation on *CDKN1B* (another known target of *IFIT3*^51^) with no pattern of increased expression of *CDKN1B* correlating with *IFIT3* expression in any cell line (Supplementary Figure S8). Importantly, there was no increase in the mRNA expression of either *CDKN1A* or *CDKN2B* in the cell lines that did not respond at this concentration and time point (JIM3 and U266), suggesting that changes in the expression of these genes could be a useful biomarker of response that should be validated *in vivo*.

## Discussion

For the first time, we have identified an association between EZH2 expression and survival in myeloma that is robust across different data sets, persists regardless of therapy used and is independent of other factors known to influence myeloma patient survival. This reinforces the importance of EZH2 expression in myeloma pathogenesis. Our findings agree with data from solid tumours and other haematological malignancies in which upregulation of EZH2 has been linked to tumour aggressiveness and poor outcomes.^22, 23, 24^ Some studies have suggested that EZH2 might be involved in transcriptional activation rather than repression and that this activation might drive tumour progression. An example of this is in prostate cancer where EZH2 acted as a co-activator for transcription factors independent of the PRC2 complex.^53^ In addition, other studies have suggested that elevated expression of EZH2 at diagnosis may be associated with a better prognosis.^54^ This would not appear to be the case in myeloma, where we have shown high EZH2 expression to be associated with features of aggressive disease, for example, increased proliferation rate and high GEP70 score. These observations are important when considering the use of EZH2 inhibitors as therapeutic strategies.

In order to gain insights into the biology of EZH2 without disruption of the PRC2 complex in myeloma, we took advantage of the recent discovery of potent and selective EZH2 inhibitors. There are currently three compounds in early-stage clinical trials across different tumour types (http://www.clinicaltrials.gov). The inhibitors were initially developed with the aim of targeting lymphomas with activating mutations but the phase I studies have seen some responses in wild-type patients and so ongoing studies continue to recruit patients with both mutant and wild-type disease.^55^ *In vitro*, the inhibitors have demonstrated activity against both the mutant and wild-type protein. The inhibitors used in this present study, EPZ005687 and UNC1999, have been well characterised in previous studies and have been shown to be specific for EZH2 with few off-target effects.^44, 47, 48^ We were, therefore, able to study the effect of inhibition of EZH2 activity in myeloma more specifically than by taking a genetic knockdown approach, which would disrupt the PRC2 complex formation as a whole and potentially yield results that might not correlate with pharmacological inhibition in patients.

Using this approach, we demonstrate the *in vitro* efficacy of EZH2 inhibition in both myeloma cell lines and in primary patient samples despite the protective effect of a modelled BM niche. Many of the cell lines and the patient samples had features of high risk disease—suggesting that EZH2 inhibition is active even in this setting. This is particularly promising given the lack of effective therapeutic options for these patients.^4^ Importantly, we identify a therapeutic index between myeloma and normal peripheral blood cells, supporting the rationale for EZH2 inhibition as a therapeutic strategy for myeloma patients.

We find that mechanistically inhibition of EZH2 function first leads to cell cycle arrest and this is followed by apoptosis. EZH2 has been shown to have a critical role in the programming of normal B-cell development and to act as a modifier defining the balance between clonal expansion and terminal differentiation, a critical balance at this stage of lymphoid development.^19^ In this setting, it is necessary for cells to come out of cell cycle in order to complete terminal differentiation and ultimately to undergo programmed cell death. Overexpression of EZH2 may function to prevent this switch by driving cell cycle progression. We went on to show that the molecular mechanism mediated via EZH2 inhibition is via either direct or indirect changes to the expression of cell cycle control genes. Results in KMS11 cells suggest that these effects might be due to focal alterations of H3K27me3 at the *CDKN1A* TSS and PROM region, but the pattern of gene expression changes supports the hypothesis that both direct and/or indirect changes in gene transcription of cell cycle inhibitors due to EZH2 inhibition, depending on the cellular context, lead to the same final common outcome (Figure 4c). In contrast, previous studies in myeloma, identifying other genes thought to be regulated by EZH2, did not identify a unifying transcriptional pattern in responding vs non-responding cell lines.^34, 35^

p21 is a member of the cip/kip family of CDK inhibitors.^56^ *CDKN1A*/p21 and other cell cycle control genes have previously been identified to be targets of EZH2/H3K27me3 in other cancers, including breast, prostate, endometrial, melanoma and non-Hodgkin's lymphoma.^26^ p21 is known to be a major target for transactivation by the tumour-suppressor gene p53 and is required for p53-dependent G1 and G2 arrest.^57, 58^ It has also previously been demonstrated to act independently of p53, as a tumour-suppressor gene in its own right,^56^ as is demonstrated by our results. p21 can not only act as a direct cell cycle inhibitor but can also supress transcription of other cell cycle control genes and promote apoptosis.^59^ EZH2 inhibition has been shown to increase p21 expression in acute myeloma leukaemia and gastric cancer^60, 61^ independent of p53 status and in melanoma where EZH2 was shown to be able to overcome p53-dependent senescence to promote the malignant phenotype.^62^ Our results support these studies and thus in myeloma inhibition of EZH2 might be used to treat patients with high-risk disease.

In myeloma, repression of other CDK inhibitors has been shown to drive tumourigenesis. *CDKN2C* (p18) is deleted in myeloma clones with 1p−,^63^ associated with a poor prognosis. *CDKN2A* (p15) and *CDKN2B* (p16) have been shown to be silenced by DNA methylation resulting in delayed cell apoptosis, poor response to chemotherapy and a shorter overall survival.^64, 65, 66^ Given the link between H3K27me3 and changes in DNA methylation,^67^ this supports our results suggesting that EZH2's role in tumourgenicity may be by repression of *CDKN1A*/p21 and *CDKN2B*/p15 via H3K27me3 silencing. This leaves unchecked the driving overexpression of cyclin D genes found in all subtypes of myeloma, leading to uncontrolled proliferation. Reversal of the H3K27me3 repression gives us the opportunity to control proliferation therapeutically.

In summary, we present evidence that EZH2 is an important therapeutic target in myeloma and suggest that clinical trials of EZH2 inhibitors enrolling myeloma patients should be considered.

## Figures and Tables

**Table 1:** Clinical and molecular features of the patients used for the analysis of EPZ005687 in primary patient CD138 selected cells

*Patient number*	*Clinical features*					*Molecular features*	
	*Age at the time of relapse (years)*	*No. of prior therapies*	*Time from first diagnosis (months)*	*Previously exposed to:*		*Translocation group/HRD*	*Copy number changes associated with risk*
				*IMiD*	*PI*		
1	57	2	36	Y	Y	None	17p−
2	56	1	27	Y	Y	t(11;14)	None
3	69	6	109	Y	Y	t(4;14) FGFR3-	1p−, 1q amp
4	50	4	33	Y	Y	t(4;14) FGFR3+	1q+
5	66	2	40	Y	Y	None	1q+
6	85	1	46	Y	N	HRD	1q+, 17p−
Median	61.5	2	38				

Abbreviations: HRD, hyperdiploid; IMiD, immunomodulatory drug; N, no; PI, proteasome inhibitor; Y, yes.
