# Supplementary material for: Overexpression of EZH2 in multiple myeloma is associated with poor prognosis and dysregulation of cell cycle control
Source: Blood Cancer J. 2017 Mar 31;7(3):e549–. doi: 10.1038/bcj.2017.27 (PMC5380911; doi:10.1038/bcj.2017.27)
Supplement: Supplementary Information [file bcj201727x1.docx]

# Supplementary Material

# Overexpression of *EZH2* in multiple myeloma is associated with poor prognosis and dysregulation of cell cycle control.

### Running title: EZH2 as a therapeutic target in Myeloma

### Authors:

Charlotte Pawlyn^1,2^, Michael D. Bright^1^, Amy F. Buros^3^, Caleb K. Stein^3^, Zoe Walters^1^, Lauren I. Aronson^1^, Fabio Mirabella^1^, John R. Jones^1,2^, Martin F. Kaiser^1,2^, Brian A. Walker^3^, Graham H. Jackson^4^, Paul A. Clarke^1^, P. Leif Bergsagel^5^, Paul Workman^1^, Marta Chesi^5^, Gareth J. Morgan^1,3^, Faith E. Davies^1,3^

1. The Institute of Cancer Research, London, UK
2. The Royal Marsden NHS Foundation Trust, London, UK
3. Myeloma Institute, University of Arkansas for Medical Sciences, Little Rock, Arkansas, USA
4. Department of Haematology, Newcastle University, Newcastle, UK
5. Mayo Clinic Arizona, Scottsdale, Arizona, USA

### Corresponding author:

Dr Charlotte Pawlyn BA, MB BChir, MRCP, PhD

The Institute of Cancer Research, 15, Cotswold Rd, London, SM2 5NG. United Kingdom

Telephone: +44 2087224130 Fax: +44 2087224432

E-mail: [charlotte.pawlyn@icr.ac.uk](mailto:charlotte.pawlyn@icr.ac.uk)

Contents

[Supplementary Material 1](#_Toc472244007)

[Overexpression of *EZH2* in multiple myeloma is associated with poor prognosis and dysregulation of cell cycle control. 1](#_Toc472244008)

[Running title: EZH2 as a therapeutic target in Myeloma 1](#_Toc472244009)

[Authors: 1](#_Toc472244010)

[Corresponding author: 1](#_Toc472244011)

[Supplementary Methods 3](#_Toc472244012)

[Expression and survival analysis – trial regimens: 3](#_Toc472244013)

[Cell viability, cell cycle and apoptosis assays 6](#_Toc472244014)

[Co-culture of BM stroma and CD138+ patient plasma cells. 7](#_Toc472244015)

[Western blotting 7](#_Toc472244016)

[Gene expression arrays 8](#_Toc472244017)

[Real-time quantitative RT-PCR (qRT-PCR) 8](#_Toc472244018)

[Chromatin immunoprecipitation-PCR (ChIP-PCR) 9](#_Toc472244019)

[Supplementary Tables 14](#_Toc472244020)

[Supplementary Table 1: Multivariate Analysis of EZH2 Expression on Clinical Outcomes in MyIX dataset 14](#_Toc472244021)

[Supplementary Table 2: Multivariate Analysis of EZH2 Expression on Clinical Outcomes in UAMS dataset 15](#_Toc472244022)

[Supplementary Table 3: Translocations and mutations present in cell lines. 16](#_Toc472244023)

[Supplementary Table 4: Gene expression of genes relevant in this study across the cell line panel used. 17](#_Toc472244024)

[Supplementary Table 5: Genes with a significant change in gene expression with EZH2 inhibition in the KMS11 cell line. (p<0.05, FDR<0.05). 18](#_Toc472244025)

[Supplementary Table 6: Genes with a significant change in expression with EZH2 inhibition in the KMM1 cell line (p<0.05). 22](#_Toc472244026)

[Supplementary Figures 26](#_Toc472244027)

[Supplementary Figure 1: Progression free survival (PFS) in the Myeloma IX and UAMS datasets 26](#_Toc472244028)

[Supplementary Figure 2: Further details of the viability analyses 27](#_Toc472244029)

[Supplementary Figure 3: Confirmatory viability experiments with a second EZH2 inhibitor UNC1999 and its negative control compound UNC2400 29](#_Toc472244030)

[Supplementary Figure 4: Confirmatory apoptosis experiments 31](#_Toc472244031)

[Supplementary Figure 5: qRT-PCR experiment with the negative control compounds UNC2400 32](#_Toc472244032)

[Supplementary Figure 6: Correlation between EZH2 and CDKN1A expression. 33](#_Toc472244033)

[Supplementary Figure 6: Further analysis of H3K27 methylation changes in response to EZH2 inhibition 34](#_Toc472244034)

[Supplementary Figure 7: Further analysis of H3K27 methylation changes in response to EZH2 inhibition 35](#_Toc472244035)

[Supplementary References: 36](#_Toc472244036)

## Supplementary Methods

### Expression and survival analysis – trial regimens:

*Myeloma IX:*

The MRC Myeloma IX study was a UK national phase III clinical trial that recruited 1970 patients between 2003 and 2009. Patients were randomised to receive either conventional chemotherapy (CVAD, cyclophosphamide, vincristine, doxorubicin, dexamethasone for those young and fit enough to proceed to autologous stem cell transplant or MP, melphalan presnisolone for older, less fit patients) or an oral triplet including the then-novel immunomodulatory agent thalidomide (CTD, cyclophosphamide, thalidomide and dexamethasone, with attenuated doses for those older and less fit). The trial had further randomisations to identify the optimal bisphosphonate for bone protection and between thalidomide or no maintenance therapy. Zolendronic acid was demonstrated to be more effective than clondronic acid both in terms of reducing skeletal events and demonstrating an independent effect on progression free and overall survival. CTD was non-inferior for PFS and OS outcomes compared to CVAD in the intensive pathway and CTDa more effective that MP in the non-intensive. Thalidomide maintenance improved PFS but not OS and was poorly tolerated. [[1-5](#_ENREF_1)]

*Total Therapy studies:*

The Total Therapy programme describes a series of clinical studies carried out at The Myeloma Institute, University of Arkansas for Medical Sciences (UAMS), Little Rock, USA. All studies have taken the approach of using all currently available myeloma-drugs in combination as part of induction therapy with the aim of destroying as many different subclones of disease as possible and preventing relapse. [[6-12](#_ENREF_6)] The drugs used in different protocols are shown in the following table with each trial having an induction, autologous stem cell transplant, consolidation and maintenance phase. These studies are limited to transplant eligible patients.

Gene expression profiling (GEP) was also performed on mRNA extracted from CD138 selected plasma cells from patients diagnosed with MGUS, SMM or MM (MGUS n=114, SMM n=163, MM n=1344) at the University of Arkansas for Medical Sciences Myeloma Institute as previously described. [[13](#_ENREF_13)] Expression between disease stages was compared with one-way ANOVA followed by Tukey’s test to look for statistically significant differences.

|  | Total Therapy 2 | | Total Therapy 3 | | Total Therapy 4 | |  | Total Therapy 5 |
| --- | --- | --- | --- | --- | --- | --- | --- | --- |
|  | TT2+ | TT- | TT3A | TT3B | TT4  lite | TT4 standard |  | TT5 |
| Induction | VAD  DCEP 1  CAD + collect  DCEP 2 + collect* | VAD  DCEP 1  CAD + collect  DCEP 2 + collect* | VTDPACE 1 + collect  VTDPACE 2 + collect* | VTDPACE 1 + collect  VTDPACE 2 + collect* | Mel10-VTDPACE 1 + collect | Mel10- VTDPACE 1 + collect  Mel10-VTDPACE 2 | **Induction** | Mel10-VTDPACE 1 + collect |
|  |  |  |  |  |  |  | **Transplant 1** | Mel80-VRDPACE |
| Transplant | Mel200 1  Mel200 2 | Mel200 1  Mel200 2 | Mel200 1  Mel200 2 | Mel200 1  Mel200 2 | fMel VTD 1  fMel VTD 2 | Mel200 1  Mel200 2 | **Interim**  **therapy** | Mel20-VTDPACE 1  Mel20-VTDPACE 2 |
| Consolidation | DPACE x 4 | DPACE x 4 | VTDPACE 1  VTDPACE 2 | VTDPACE 1  VTDPACE 2 | VTDPACE 1 | VTDPACE 1  VTDPACE 2 | **Transplant 2** | Mel80-VRDPACE |
| Maintenance | Year 1: Dex + IFN + Thal  Year 2-3: IFN+ Thal | Year 1: Dex + IFN  Year 2-3: IFN | Year 1: VTD  Year 2-3: Thal + dex | Year 1-3: VRD | Year 1-3: VRD | Year 1-3: VRD | **Maintenance** | Year 1-3: VRD |

**Total Therapy trial treatment outlines.** VAD = vincristine, doxorubicin, dexamethasone. DCEP = dexamethasone, cyclophosphamide, etoposide and cisplatin. CAD = cyclophosphamide, adriamycin and dexamethasone. Mel = melphalan, DPACE = dexamethasone, cisplatin, adriamycin, cyclophosphamide, etoposide. Dex = dexamethasone. IFN = interferon. thal = thalidomide. VTDPACE = bortezomib, dexamethasone, thalidomide, cisplatin, adriamycin, cyclophosphamide, etoposide. VTD = bortezomib, thalidomide, dexamethasone. VRD = bortezomib, lenalidomide, dexamethasone. VRDPACE – bortezomib, lenalidomide, dexamethasone, cisplatin, adriamycin, cyclophosphamide, etoposide. fMel = fractionated melphalan

*if insufficient cells collected at first collection

### Cell viability, cell cycle and apoptosis assays

Cell lines were purchased from American Type Culture Collection (ATCC) RPMI8226, U266, MM1S, HS-5 or were a gift from Professor H. Johnsen (Århus University Hospital, Denmark) KMS11, KMS12BM, LP1, KMM1.

Inhibition of proliferation in cell lines was measured using the in vitro WST-1 colorimetric assay according to the manufacturer’s instructions (Roche). For ≤72 hour assays, cells were seeded in 96-well plates at a density of 10,000 cells per well in a total volume of 100ul phenol-red free media. Compound or vector control (DMSO) was added after 2-4 hours at the concentration indicated. Plates/flasks were incubated in a humidified incubator in 5% CO_2_ for 24 to 72 hours at 37^◦^C. Subsequently 10ul WST-1 solution was added to each well. After a further 4hrs absorbance at 420nm and 630nm was measured using the Epoch Microplate Spectrophotometer (BioTek). For 6 day assays 40,000 cells/ml (final volume 5mls) were seeded in a T25 flask and compound or DMSO added at the indicated concentration. At day 3 the media and compound were replaced. At day 6 cells were resuspended (after scrapping for adherent cell lines) and 100ul of cell/media per well placed in a 96 well plate in triplicate before proceeding with the assay as described above.

For all other assays 3 and 6 days incubations were carried out as follows: 40,000 cells/ml (final volume 5mls) were seeded in a T25 flask and compound or DMSO added at the indicated concentration. If proceeding to 6 days, at day 3 the media and compound were replaced.

Flow cytometry was used to look for evidence of apoptosis. After harvesting cells were resuspended in Annexin Binding Buffer 1x (BD Biosciences) and stained with 5ul Annexin V – APC (BD Biosciences) and, after 15 mins, additionally with 5ul PI (BD Biosciences). Greater than 10,000 cells were analysed per sample using a BD LSRII flow cytometer. The proportion of Annexin plus Annexin and PI positive cells (compared to DMSO control) was calculated.

To demonstrate caspase activity the Caspase-Glo® 3/7 luminescent assay was used according to manufacturer’s instructions (Promega). At day 6 cells were resuspended (after scrapping for adherent cell lines) and counted. 0.2x10^6 cells/ml were plated in a 96 well plate in triplicate (100ul per well). 50ul of Caspase-Glo® was added and the luminescene read after 1 hr incubation at room temperature using a Mithras LB940 Multimode Microplate Reader (Berthold). Luminescence increases proportional to increasing activity of caspase 3/7 due to cleavage of the luminogenic caspase-3/7 substrate which contains the tetrapeptide sequence DEVD. Relative luminescence was calculated compared to DMSO control.

For cell cycle analysis at the desired time point cells were washed in PBS prior to resuspension in 70% ice cold ethanol. They were incubated at 4^◦^C for 15 mins. After washing they were incubated with RNase and PI for 25 mins at 37^◦^C prior to analysis by flow cytometry.

Flow cytometry data was analysed using BD FACSDiva^TM^ software and/or FlowJo©

Co-culture of BM stroma and CD138+ patient plasma cells.

0.01x106 HS5-GFP cells were plated in 250ul RPMI medium in a 48 well plate and incubated at 37◦C in a humidified gas chamber for 4 hours. 0.05x106 CD138+ primary myeloma cells or cell lines were then added in RPMI to give a final volume of 500ul. Compound or control was added and plates incubated for 72 hours.

### Western blotting

Cell pellets were lysed in RIPA buffer supplemented to 1% SDS. Samples for histone blotting were sonicated for 10 mins at 4^◦^C after suspension in RIPA buffer. Protein was quantified using a BCA protein assay (Pierce). Equal amounts of protein were run on a 4-12% Bis-Tris gel (NuPage, Novex) in MOPS SDS running buffer (NuPage). Gels were transferred to an Immobilon-P membrane using a wet transfer system. The membrane was blocked with 5% bovine serum albumin for 1 hour. Primary antibodies were incubated with the membrane over night at 4^◦^C, the membrane was washed and then incubated with secondary antibody for 1 hr at room temperature. The membrane was then incubated with ECL (GeneFlow) for 1 minute before exposure using either film or a FlourChem E imager (Proteinsimple).

Antibodies and dilutions used:

| Protein | M.W. (kDa) | Primary Ab | Dilution  (in 5% BSA) | Secondary Ab | Dilution  (in 5% BSA) |
| --- | --- | --- | --- | --- | --- |
| PARP and cleaved PARP | 116,  89 | Cell Signalling Technologies #9532 | 1:1000 | Anti-rabbit | 1:10,000 |
| p15 | 15 | Abcam Ab53034 | 1:500 | Anti-rabbit | 1:10,000 |
| p21 | 21 | Cell Signalling Technologies #2947 | 1:500 | Anti-rabbit | 1:10,000 |
| H3K27me1 | 17 | Active Motif AM61015 | 1:2000 | Anti-mouse | 1:10,000 |
| H3K27me2 | 17 | Active Motif AM61435 | 1:2000 | Anti-mouse | 1:10,000 |
| H3K27me3 | 17 | Active Motif AM61017 | 1:2000 | Anti-mouse | 1:10,000 |
| H3K36me2 | 17 | Active Motif AM61019 | 1:2000 | Anti-mouse | 1:10,000 |
| EZH2 | 91 | BD biosciences 612667 | 1:1000 | Anti-mouse | 1:10,000 |
| Total H3 | 17 | Active Motif AM39763 | 1:2000 | Anti-mouse | 1:10,000 |
| Actin | 42 | Sigma A5441-2ML | 1:5000 | Anti-mouse | 1:10,000 |

Anti-mouse: IgG HRP-linked anti-mouse antibody New England Biotech 7076S

Anti-rabbit: IgG HRP-linked anti-rabbit antibody New England Biotech 7074S

### Gene expression arrays

40,000 cells/ml (final volume 5mls) were seeded in a T25 flask and EPZ005687 or DMSO added at the indicated concentration. At day 3 the media and compound were replaced. At day 6 cell pellets were frozen and mRNA extraction and processing performed by the Gene Expression Core Facility at the Myeloma Institute, University of Arkansas for Medical Sciences as previously described [[13](#_ENREF_13)] using Affymetrix HG U133 plus 2 arrays. These were repeated in triplicate in KMS11 and duplicate in KMM1. The resulting Cel files were analysed using Partek® software with the Robust Multi-array Average (RMA) method. This includes background correction, which normalises the distribution across arrays and, quantile normalisation to correct for array biases. There is then a calculation of probe level intensity. Median Polish is then used for probeset summarisation which gives a single intensity value for each probeset. Probesets with a greater than 2 fold difference in expression that was statistically significant following ANOVA and Fisher’s Least Significant Difference analysis at p<0.05 were considered significant with FDR correction for KMS11 and without for KMM1.

### Real-time quantitative RT-PCR (qRT-PCR)

Cell pellets were lysed in RLT buffer and homogenised using QIAshredder (Qiagen). RNA was extracted using RNeasy Plus mini kit (Qiagen) according to manufactures instruction, quantified using a NanoDrop spectrophotometer and the quantity normalised before reverse transcription performed using High-Capacity cDNA Reverse Transcription Kit (Invitrogen). qRT-PCR was performed using Taqman reagents (Life Technologies) to determine the comparative Ct (compared to DMSO control) for the indicated products using a 7500 Fast PCR system (Applied Biosystems). Taqman assay IDs used were:

| Gene | Taqman assay ID |
| --- | --- |
| CDKN1A | Hs00355782_m1 |
| CDKN1B | Hs01597588_m1 |
| CDKN2A | Hs00923894_m1 |
| CDKN2B | Hs00793225_m1 |
| MYC | Hs00153408_m1 |
| IRF4 | Hs01056533_m1 |
| IFIT3 | Hs01922752_s1 |
| SKP2 | Hs01021864_m1 |
| EZH2 | Hs01016789_m1 |
| GAPDH | Hs99999905_m1 |

### Chromatin immunoprecipitation-PCR (ChIP-PCR)

ChIP-PCR was performed using the ChIP-IT Express Enzymatic kit (Active Motif) with optimisations/modifications. Samples at 6 days were fixed in formaldehyde (final concentration 1%) for 6 mins before quenching the reaction with glycine. After washing, cell pellets were snap frozen (dry-ice) and stored at -80^◦^C till used. Pellets were thawed and resuspended in lysis buffer for 40 mins at 4^◦^C. Samples were lysed using a dounce for 150 strokes and the nuclei pelleted. The nuclei were resuspended in digestion buffer and incubated at 37^◦^C for 5 mins. The resulting chromatin was incubated with enzymatic shearing cocktail (2.5ul per 50ul chromatin) for 10 mins at 37^◦^C before the reaction was halted with EDTA. Samples were then sonicated for 10 mins at 4^◦^C. (These were the best conditions identified following several optimisation experiments). The sheared chromatin was quantified using the nanodrop and shearing confirmed on a Tapestation (Agilent). Chromatin concentration was normalised by dilution with digestion buffer, an input sample taken and an equal amount (1.5ug) used for each ChIP reaction. The ChIP reaction was then set up with modifications to the protocol including 1) incubation of the protein G beads with mouse bridging antibody (Active Motif) for 1 hour prior to washing and use in the ChIP, 2) Addition of salmon sperm DNA to the ChIP reaction at a final concentration of 20ug/ul. Two parallel ChIP reactions were set up with each chromatin sample, one for H3K27me3 (Active Motif 61017) and one for an isotype control antibody (Mouse IgG1 isotype control, NEB 5415S) used at the same final concentration as H3K27me3. The ChIP reaction was incubated overnight at 4^◦^C on a rotator.

The following day, samples were washed and the chromatin eluted and reverse cross linked for both the antibody pull down and input samples. The DNA was cleaned (Zymo ChIP DNA Clean and Concentrator) and eluted in 100ul of elution buffer. 4ul was used per PCR reaction. PCR was performed using Power SYBR ® Green Real-Time PCR Master Mix (Thermo Fisher) on an Applied Biosystems® 7500 Fast Real-Time PCR System. Primers were designed using Primer Express ® (Thermo Fisher) at the transcriptional start site and the promotor region of each gene of interest at areas that appeared marked with regulatory/promoter elements using tracks from ENCODE on UCSC genome browser. Additional primers were deigns to regions approximately 5Kb upstream of the TSS and not marked by ENCODE tracks. Primers were optimised using standard curves at different concentrations and genomic DNA from KMS11 to find the concentration with the best efficiency and to check there was a single melt curve peak. Primers were used in the experimental plates at their optimised concentrations. Positive and negative control primer sets were also run (Active Motif).

| Region | Fw | Rw | Optimal conc. |
| --- | --- | --- | --- |
| *CDKN2B* TSS | CTAGGCGCTTTTTCCCAGAA | GCTTTTCCTGGCGCTCAAG | 500nM |
| *CDKN2B* PROM | TGCAGAGCTGTCGCTTTCAG | GTGACCGAGAGAAAGTCATTCAAA | 900nM |
| *CDKN2B* -5Kb | AAGGAAGTCTGCCTATATGGGTTATC | TCCGAATTCAGTTGGGTTTGT | 250nM |
| *IFIT3* PROM | CATATCCAGCTTCCCCTTCACA | TTTTGGCCAAGGGCATTC | 900nM |
| *IFIT3* -5Kb | CCCACCACCGTGCTTCTAGA | CAAGGCTTGCAGAAGTCTCCTT | 500nM |
| *CDKN1A* PROM | CTTCTCTGAGCCCCAGTTTCC | GGATTTGACGAGTGAGTTGTCTGT | 500nM |
| *CDKN1A* TSS | CGCGAGGATGCGTGTTC | CATTCACCTGCCGCAGAAA | 900nM |
| *CDKN1A* -5Kb | CCAATATGTGTTCAGGGCTTTAGA | ACCAGGTATCTGGGCCTCACT | 900nM |
| Negative control | Active motif negative control primer set 3, *ACTB* | | As AM protocol |
| Positive control | Active motif positive control primer set, *MYT1* | | As AM protocol |

A standard curve for each primer pair was run on every plate using the input sample of the DMSO treated samples and ten-fold dilutions of this. Input samples were then diluted 1:10 and ChIP samples used neat. % input calculations for each sample were made using the standard curve and the comparative CT method. All samples were run in duplicate and the mean taken for each experiment. Each ChIP-PCR was repeated with a second complete biological replicate.

The location of the product for each primer set is shown in the following diagrams (images from UCSC genome browser):

*CDKN2B* TSS:


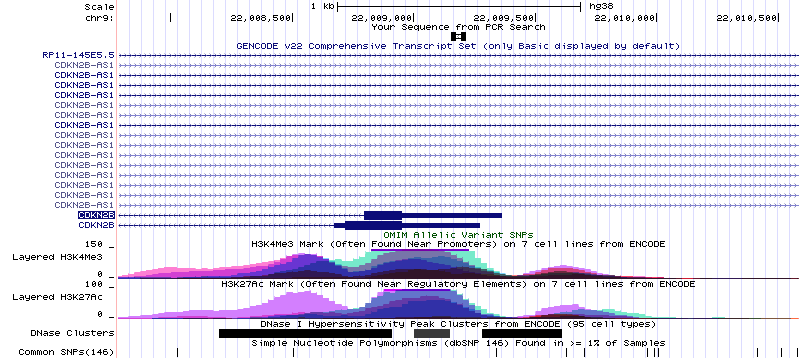


*CDKN2B* PROM:


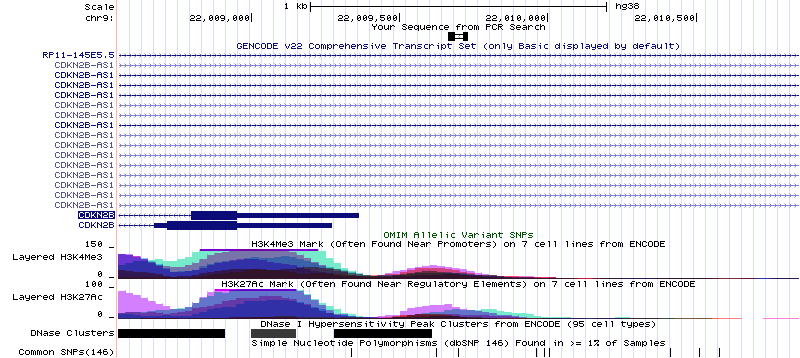


*CDKN2B* -5Kb:


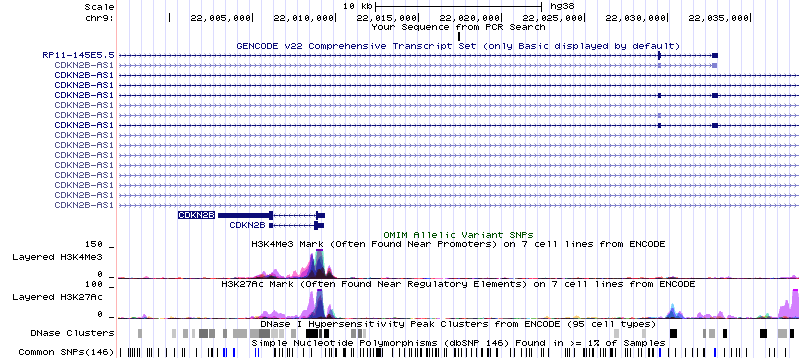


*IFIT3* PROM:


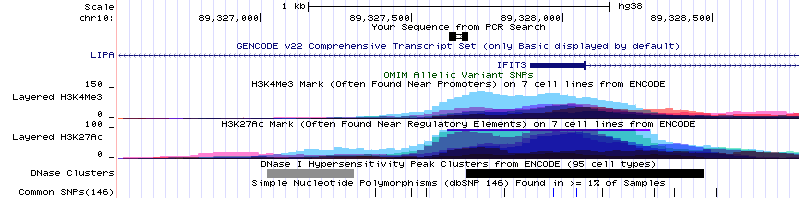


*IFIT3* -5Kb:


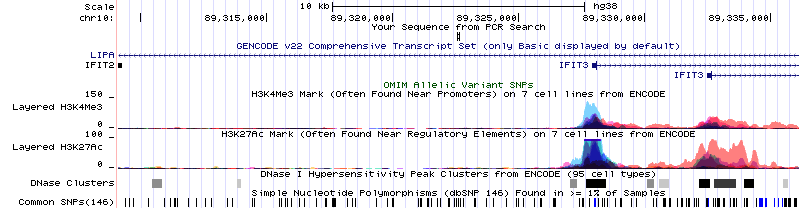


*CDKN1A* PROM:


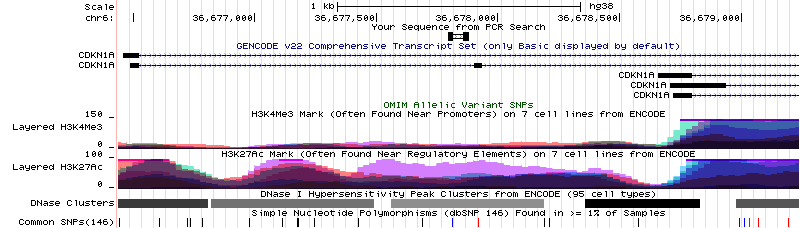


*CDKN1A* TSS:


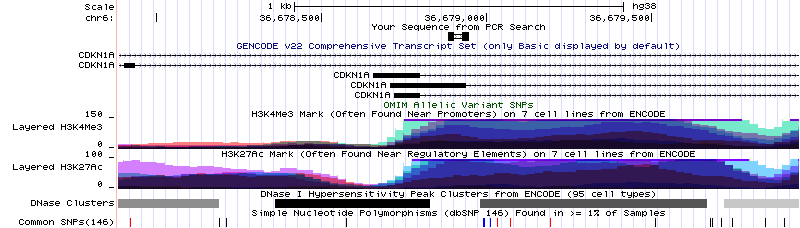


*CDKN1A* -5Kb:


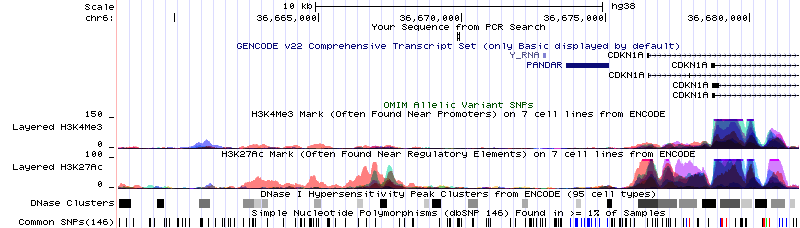


## Supplementary Tables

### Supplementary Table 1: Multivariate Analysis of EZH2 Expression on Clinical Outcomes in MyIX dataset

All variables were included in a backwards selection model. The significance threshold for remaining in the model at each stage was set at 0.05. Variables that remained significant at the end of the selection model are shown below. 159 patients (with values for all variables) were included in the analysis.

|  | Overall Survival | |
| --- | --- | --- |
| Variable | **HR (95% CI)** | **p-value** |
| Sex  (M vs F) | - | - |
| ISS  (II/III vs I) | 2.369 (1.360, 4.126) | 0.002 |
| t(11;14)  (present vs not) | - | - |
| Bone disease  (present vs not) | - | - |
| HRD  (present vs not) | - | - |
| Any ad translocation  (present vs not) | 1.873 (1.226, 2.861) | 0.004 |
| Del17p  (present vs not) | - | - |
| 1q+  (present vs not) | - | - |
| Chemotherapy  (thalidomide vs not) | - | - |
| Pathway  (Non-intensive vs intensive) | 2.116 (1.443, 3.101) | <0.001 |
| High *EZH2*  (>8.24 vs <8.24) | 2.064 (1.257, 3.390) | 0.004 |

HR – Hazard Ratio, 95% CI- 95% Confidence Interval, p-value from multivariate analysis

ad translocation = t(4;14), t(14;16) or t(14;20)

### Supplementary Table 2: Multivariate Analysis of EZH2 Expression on Clinical Outcomes in UAMS dataset

All variables in the Univariate analysis were included in a backwards selection model. Significance threshold for remaining in the model was set at 0.05.

|  | Overall Survival | |
| --- | --- | --- |
| Variable | **HR (95%) CI** | **p-value** |
| Age ≥ 65 years | 1.47 (1.22, 1.78) | <.0001 |
| ISS (I vs II/III) | 1.56 (1.29, 1.89) | <.0001 |
| Creatinine ≥ 2 mg/dL |  |  |
| LDH ≥190 U/L | 1.42 (1.18, 1.72) | 0.0002 |
| GEP70 High Risk | 2.46 (1.92, 3.14) | <.0001 |
| High EZH2 | 1.45 (1.15, 1.81) | 0.0016 |

HR – Hazard Ratio, 95% CI- 95% Confidence Interval, p-value from Wald Test in Cox Regression

### Supplementary Table 3: Translocations and mutations present in cell lines.

|  | KMS11 | JIM3 | MM1.S | LP1 | KMS12BM | RPMI  8226 | KMM1 | U266 |
| --- | --- | --- | --- | --- | --- | --- | --- | --- |
| Structural abnormality | | | | | | | | |
| Trans-location | t(4;14) t(14;16) | t(4;14) | t(14;16) | t(4;14) | t(11;14) | t(14;16) | t(6;14) | t(11;14) |
| Mutation | | | | | | | | |
| *EZH2* |  |  |  |  |  |  |  |  |
| *KDM6A* |  |  |  | p.Q593* p.Q541X | HD | p.Q527P |  |  |
| *MMSET* |  |  | p.E1099K |  |  |  |  |  |
| *ARID1A* |  |  |  |  |  | p.P120S |  |  |
| *TP53* | HD | p.R273C hom |  | p.E286K hom | p.R337L hom | p.E285K hom | p.S241F het | p.A161T hom |
| High risk features? | | | | | | | | |
|  | Y | Y | Y | Y | N | Y | N | N |

The cell line features of factors previously demonstrated to be relevant to EZH2 inhibition, *TP53* and risk status are shown in the table. (details from Broad CCLE, MMRF Myeloma Cell Line Characterization Data repository and [[14](#_ENREF_14)]). HD = heterozygous deletion, hom = homozygous mutation, het = heterozygous mutation. Where the field is blank no mutation was identified.

### Supplementary Table 4: Gene expression of genes relevant in this study across the cell line panel used.

|  | **KMS11** | **JIM3** | **MM1.S** | **LP1** | **KMS12**  **BM** | **RPMI**  **8226** | **KMM1** | **U266** |
| --- | --- | --- | --- | --- | --- | --- | --- | --- |
| Gene expression - MMRF (RNA seq - FPKBM) | | | | | | | | |
| *EZH2* | 64.35 | 33.35 | 49.78 | 75.18 | 60.12 | 40.45 | 19.57 | 67.37 |
| *MMSET* | 141.36 | 271.25 | 60.73 | 120.22 | 61.88 | 36.44 | 49.85 | 49.27 |
| *KDM6A* | 16.92 | 12.96 | 11.76 | 7.25 | 20.48 | 1.39 | 27.99 | 19.87 |
| *ARID1A* | 40.42 | 51.53 | 44.80 | 55.46 | 31.48 | 27.39 | 35.00 | 39.99 |
| *KRAS* | 29.57 | 60.79 | 35.86 | 52.87 | 41.65 | 51.70 | 57.68 | 35.26 |
| *NRAS* | n/a | n/a | n/a | n/a | n/a | n/a | n/a | n/a |
| *CDKN2B* | 2.63 | 0.07 | 0.16 | 20.97 | 10.67 | 0.88 | 15.52 | 10.84 |
| *CDKN1A* | 0.46 | 0.28 | 23.56 | 1.12 | 1.63 | 45.82 | 4.85 | 52.07 |
| Gene expression - CCLE (mRNA expression array, log2) | | | | | | | | |
| *EZH2* | 11.13 | n/a | 11.08 | 8.61 | 10.59 | 10.36 | 10 | 10.36 |
| *MMSET* | 9.74 | n/a | 8.56 | 9.6 | 8.28 | 7.69 | 8.51 | 7.71 |
| *KDM6A* | 7.35 | n/a | 7.83 | 6.32 | 7.74 | 3.69 | 7.5 | 6.79 |
| *ARID1A* | 9.05 | n/a | 9.54 | 9.79 | 8.8 | 8.97 | 8.9 | 8.76 |
| *KRAS* | 11.4 | n/a | 11.06 | 11.65 | 11.03 | 11.45 | 11.19 | 10.72 |
| *NRAS* | 9.27 | n/a | 9.16 | 9.53 | 10.01 | 9.04 | 10.59 | 10.42 |
| *CDKN2B* | 4.61 | n/a | 3.76 | 5.26 | 4.46 | 3.89 | 5.37 | 5.09 |
| *CDKN1A* | 4.52 | n/a | 8.16 | 4.46 | 4.91 | 7.50 | 4.73 | 8.41 |

Details from Multiple Myeloma Research Foundation (MMRF) Cell Line Data Repository and Broad Cancer Cell Line Encyclopedia (CCLE). n/a indicates that the test was not done in that dataset. The intensity of green shading is relative to the comparative expression of each gene across the 8 cell lines. FPKBM – fragments per kilobase matched.

### Supplementary Table 5: Genes with a significant change in gene expression with EZH2 inhibition in the KMS11 cell line. (p<0.05, FDR<0.05).

A negative fold changes indicates an increase in gene expression with EZH2 inhibition. ‘---‘ = probeset not recognised.

| Column ID | Gene Symbol | Gene Name | p-value (DMSO vs. EZH2 inhibitor) | Fold-Change (DMSO vs. EZH2 inhibitor) |
| --- | --- | --- | --- | --- |
| 239468_at | MKX | mohawk homeobox | 9.64E-05 | -4.69356 |
| 205114_s_at | CCL3 /// CCL3L1 /// CCL3L3 | chemokine (C-C motif) ligand 3 /// chemokine (C-C motif) ligand 3-like 1 /// chemokine | 0.000114818 | -4.45077 |
| 201669_s_at | MARCKS | myristoylated alanine-rich protein kinase C substrate | 1.21E-05 | -3.95147 |
| 205542_at | STEAP1 | six transmembrane epithelial antigen of the prostate 1 | 4.22E-05 | -3.88737 |
| 220014_at | PRR16 | proline rich 16 | 0.000100297 | -3.6433 |
| 201670_s_at | MARCKS | myristoylated alanine-rich protein kinase C substrate | 0.000327875 | -3.21058 |
| 226517_at | BCAT1 | branched chain amino-acid transaminase 1, cytosolic | 0.000202499 | -3.00234 |
| 217764_s_at | RAB31 | RAB31, member RAS oncogene family | 1.21E-06 | -2.80908 |
| 204897_at | PTGER4 | prostaglandin E receptor 4 (subtype EP4) | 0.000292292 | -2.76252 |
| 217762_s_at | RAB31 | RAB31, member RAS oncogene family | 2.17E-07 | -2.76105 |
| 221523_s_at | RRAGD | Ras-related GTP binding D | 2.63E-06 | -2.70501 |
| 236313_at | CDKN2B | cyclin-dependent kinase inhibitor 2B (p15, inhibits CDK4) | 0.000490694 | -2.66566 |
| 229973_at | ERICH3 | glutamate-rich 3 | 1.25E-05 | -2.63942 |
| 209524_at | HDGFRP3 | hepatoma-derived growth factor, related protein 3 | 0.000142138 | -2.60752 |
| 217763_s_at | RAB31 | RAB31, member RAS oncogene family | 1.90E-05 | -2.4938 |
| 225285_at | BCAT1 | branched chain amino-acid transaminase 1, cytosolic | 0.000180417 | -2.4522 |
| 236099_at | --- | --- | 0.000328768 | -2.45031 |
| 226534_at | KITLG | KIT ligand | 1.11E-05 | -2.40677 |
| 227425_at | REPS2 | RALBP1 associated Eps domain containing 2 | 0.000111425 | -2.32696 |
| 220253_s_at | LRP12 | low density lipoprotein receptor-related protein 12 | 1.17E-06 | -2.32119 |
| 226865_at | PLXDC2 | plexin domain containing 2 | 4.89E-05 | -2.25942 |
| 226225_at | MCC | mutated in colorectal cancers | 0.000393934 | -2.20352 |
| 203510_at | MET | MET proto-oncogene, receptor tyrosine kinase | 0.000483785 | -2.20292 |
| 241902_at | MKX | mohawk homeobox | 0.000165061 | -2.14801 |
| 219631_at | LRP12 | low density lipoprotein receptor-related protein 12 | 1.61E-06 | -2.14437 |
| 205501_at | PDE10A | phosphodiesterase 10A | 6.80E-05 | -2.10843 |
| 226884_at | LRRN1 | leucine rich repeat neuronal 1 | 4.22E-06 | -2.0934 |
| 212724_at | RND3 | Rho family GTPase 3 | 4.73E-05 | -2.08633 |
| 223218_s_at | NFKBIZ | nuclear factor of kappa light polypeptide gene enhancer in B-cells inhibitor, zeta | 7.43E-06 | -2.00701 |
| 221524_s_at | RRAGD | Ras-related GTP binding D | 1.50E-05 | -2.0026 |
| 203642_s_at | COBLL1 | cordon-bleu WH2 repeat protein-like 1 | 6.87E-05 | -1.98761 |
| 203710_at | ITPR1 | inositol 1,4,5-trisphosphate receptor, type 1 | 1.47E-05 | -1.98105 |
| 203290_at | HLA-DQA1 | major histocompatibility complex, class II, DQ alpha 1 | 0.00015466 | -1.96995 |
| 227623_at | CACNA2D1 | calcium channel, voltage-dependent, alpha 2/delta subunit 1 | 3.35E-05 | -1.96584 |
| 205003_at | DOCK4 | dedicator of cytokinesis 4 | 1.20E-06 | -1.95836 |
| 203021_at | SLPI | secretory leukocyte peptidase inhibitor | 2.15E-05 | -1.92975 |
| 236335_at | GUCY1A2 | guanylate cyclase 1, soluble, alpha 2 | 0.000186518 | -1.92935 |
| 205645_at | REPS2 | RALBP1 associated Eps domain containing 2 | 7.40E-05 | -1.91204 |
| 212843_at | NCAM1 | neural cell adhesion molecule 1 | 2.46E-05 | -1.90195 |
| 238623_at | RP3-428L16.2 | --- | 3.53E-05 | -1.87489 |
| 201117_s_at | CPE | carboxypeptidase E | 2.36E-05 | -1.86254 |
| 228731_at | GUCY1A2 | guanylate cyclase 1, soluble, alpha 2 | 0.000884302 | -1.86216 |
| 227276_at | PLXDC2 | plexin domain containing 2 | 0.000634596 | -1.85197 |
| 228438_at | LOC100132891 | uncharacterised LOC100132891 | 8.65E-05 | -1.8335 |
| 205890_s_at | GABBR1 /// UBD | gamma-aminobutyric acid (GABA) B receptor, 1 /// ubiquitin D | 0.00041961 | -1.8333 |
| 201939_at | PLK2 | polo-like kinase 2 | 0.000204787 | -1.82925 |
| 242571_at | REPS2 | RALBP1 associated Eps domain containing 2 | 0.000342406 | -1.80211 |
| 238914_at | DCC | DCC netrin 1 receptor | 0.000160269 | -1.79905 |
| 227341_at | BEND7 | BEN domain containing 7 | 8.60E-05 | -1.79784 |
| 226853_at | BMP2K | BMP2 inducible kinase | 7.43E-05 | -1.79289 |
| 204422_s_at | FGF2 | fibroblast growth factor 2 (basic) | 0.000517681 | -1.78546 |
| 232060_at | ROR1 | receptor tyrosine kinase-like orphan receptor 1 | 2.68E-05 | -1.7837 |
| 227312_at | SNTB2 | syntrophin, beta 2 (dystrophin-associated protein A1, 59kDa, basic component 2) | 0.000148409 | -1.77111 |
| 201468_s_at | NQO1 | NAD(P)H dehydrogenase, quinone 1 | 4.17E-05 | -1.76575 |
| 206290_s_at | RGS7 | regulator of G-protein signaling 7 | 9.04E-06 | -1.76534 |
| 216693_x_at | HDGFRP3 | hepatoma-derived growth factor, related protein 3 | 0.000199459 | -1.76323 |
| 238592_at | --- | --- | 0.000296639 | -1.7616 |
| 236738_at | C3orf80 | chromosome 3 open reading frame 80 | 1.22E-05 | -1.75884 |
| 227370_at | FAM171B | family with sequence similarity 171, member B | 0.000212971 | -1.75154 |
| 219926_at | POPDC3 | popeye domain containing 3 | 0.000200414 | -1.73201 |
| 205206_at | KAL1 | Kallmann syndrome 1 sequence | 1.51E-05 | -1.72934 |
| 226685_at | SNTB2 | syntrophin, beta 2 (dystrophin-associated protein A1, 59kDa, basic component 2) | 2.63E-06 | -1.72901 |
| 201471_s_at | SQSTM1 | sequestosome 1 | 4.24E-05 | -1.72715 |
| 225798_at | JAZF1 | JAZF zinc finger 1 | 6.33E-05 | -1.71566 |
| 235146_at | TMCC3 | transmembrane and coiled-coil domain family 3 | 9.54E-05 | -1.71311 |
| 211729_x_at | BLVRA | biliverdin reductase A | 3.98E-06 | -1.71129 |
| 207076_s_at | ASS1 | argininosuccinate synthase 1 | 4.00E-05 | -1.70707 |
| 203773_x_at | BLVRA | biliverdin reductase A | 2.64E-07 | -1.7001 |
| 205943_at | TDO2 | tryptophan 2,3-dioxygenase | 0.000587861 | -1.68883 |
| 209526_s_at | HDGFRP3 | hepatoma-derived growth factor, related protein 3 | 0.000328048 | -1.68021 |
| 202932_at | YES1 | YES proto-oncogene 1, Src family tyrosine kinase | 0.00043182 | -1.64859 |
| 228494_at | PPP1R9A | protein phosphatase 1, regulatory subunit 9A | 0.000407273 | -1.63542 |
| 205315_s_at | SNTB2 | syntrophin, beta 2 (dystrophin-associated protein A1, 59kDa, basic component 2) | 0.000562703 | -1.63446 |
| 228266_s_at | HDGFRP3 | hepatoma-derived growth factor, related protein 3 | 0.000181445 | -1.63385 |
| 223248_at | HSDL1 | hydroxysteroid dehydrogenase like 1 | 2.14E-06 | -1.62988 |
| 212820_at | DMXL2 | Dmx-like 2 | 0.000152746 | -1.62371 |
| 203771_s_at | BLVRA | biliverdin reductase A | 0.000177258 | -1.61611 |
| 202933_s_at | YES1 | YES proto-oncogene 1, Src family tyrosine kinase | 6.69E-05 | -1.6118 |
| 226438_at | SNTB1 | syntrophin, beta 1 (dystrophin-associated protein A1, 59kDa, basic component 1) | 0.000137264 | -1.60608 |
| 201467_s_at | NQO1 | NAD(P)H dehydrogenase, quinone 1 | 0.000330916 | -1.6017 |
| 221210_s_at | NPL | N-acetylneuraminate pyruvate lyase (dihydrodipicolinate synthase) | 0.000764932 | -1.59896 |
| 212956_at | TBC1D9 | TBC1 domain family, member 9 (with GRAM domain) | 1.68E-06 | -1.59668 |
| 204944_at | PTPRG | protein tyrosine phosphatase, receptor type, G | 0.000370323 | -1.59624 |
| 225800_at | JAZF1 | JAZF zinc finger 1 | 0.000137485 | -1.59156 |
| 212097_at | CAV1 | caveolin 1, caveolae protein, 22kDa | 0.000226957 | -1.58982 |
| 201212_at | LGMN | legumain | 0.000218717 | -1.58546 |
| 203324_s_at | CAV2 | caveolin 2 | 0.000301789 | -1.58224 |
| 204998_s_at | ATF5 | activating transcription factor 5 | 0.000684352 | -1.58115 |
| 208949_s_at | LGALS3 | lectin, galactoside-binding, soluble, 3 | 0.000283561 | -1.57685 |
| 203323_at | CAV2 | caveolin 2 | 0.000668098 | -1.57528 |
| 1559170_at | ANKRD20A5P /// ANKRD20A5P | Homo sapiens ankyrin repeat domain 20 family, member A5, pseudogene (ANKRD20A5P), non-c | 0.000832421 | -1.56632 |
| 205544_s_at | CR2 | complement component (3d/Epstein Barr virus) receptor 2 | 8.53E-06 | -1.56155 |
| 223638_at | NBPF3 | neuroblastoma breakpoint family, member 3 | 3.10E-05 | -1.55914 |
| 211368_s_at | CASP1 | caspase 1, apoptosis-related cysteine peptidase | 0.000526471 | -1.55614 |
| 223405_at | NPL | N-acetylneuraminate pyruvate lyase (dihydrodipicolinate synthase) | 0.000782977 | -1.54852 |
| 210519_s_at | NQO1 | NAD(P)H dehydrogenase, quinone 1 | 4.54E-05 | -1.54739 |
| 228949_at | WLS | wntless Wnt ligand secretion mediator | 0.000178478 | -1.54239 |
| 226066_at | MITF | microphthalmia-associated transcription factor | 4.95E-06 | -1.53473 |
| 226145_s_at | FRAS1 | Fraser extracellular matrix complex subunit 1 | 5.91E-05 | -1.53349 |
| 223217_s_at | NFKBIZ | nuclear factor of kappa light polypeptide gene enhancer in B-cells inhibitor, zeta | 0.000170726 | -1.5269 |
| 238332_at | ANKRD29 | ankyrin repeat domain 29 | 8.84E-05 | -1.52661 |
| 225252_at | SRXN1 | sulfiredoxin 1 | 0.000351683 | -1.51163 |
| 232235_at | DSEL | dermatan sulfate epimerase-like | 0.00020335 | -1.5046 |
| 204364_s_at | REEP1 | receptor accessory protein 1 | 0.0003488 | -1.50113 |
| 209485_s_at | OSBPL1A | oxysterol binding protein-like 1A | 0.000206319 | -1.50062 |
| 1555292_at | STRIP2 | striatin interacting protein 2 | 0.000598113 | -1.50008 |
| 200675_at | CD81 | CD81 molecule | 2.37E-05 | 1.50078 |
| 204148_s_at | POMZP3 /// ZP3 | POM121 and ZP3 fusion /// zona pellucida glycoprotein 3 (sperm receptor) | 0.000133893 | 1.50088 |
| 221011_s_at | LBH | limb bud and heart development | 9.39E-06 | 1.50404 |
| 212057_at | GSE1 | Gse1 coiled-coil protein | 0.000707034 | 1.50648 |
| 205098_at | CCR1 | chemokine (C-C motif) receptor 1 | 0.000189848 | 1.50817 |
| 224774_s_at | NAV1 | neuron navigator 1 | 9.86E-05 | 1.50894 |
| 203634_s_at | CPT1A | carnitine palmitoyltransferase 1A (liver) | 0.000451426 | 1.51585 |
| 219862_s_at | NARF | nuclear prelamin A recognition factor | 9.78E-06 | 1.52216 |
| 211555_s_at | GUCY1B3 | guanylate cyclase 1, soluble, beta 3 | 0.000139731 | 1.52727 |
| 33304_at | ISG20 | interferon stimulated exonuclease gene 20kDa | 0.000110618 | 1.53027 |
| 227915_at | ASB2 | ankyrin repeat and SOCS box containing 2 | 0.000767191 | 1.53358 |
| 1552634_a_at | ZNF101 | zinc finger protein 101 | 0.000467444 | 1.53517 |
| 204820_s_at | BTN3A2 /// BTN3A3 | butyrophilin, subfamily 3, member A2 /// butyrophilin, subfamily 3, member A3 | 0.000254518 | 1.53532 |
| 215785_s_at | CYFIP2 | cytoplasmic FMR1 interacting protein 2 | 7.25E-06 | 1.54028 |
| 240449_at | ZNF341 | zinc finger protein 341 | 0.000917744 | 1.54719 |
| 205659_at | HDAC9 | histone deacetylase 9 | 2.54E-06 | 1.54846 |
| 212613_at | BTN3A2 | butyrophilin, subfamily 3, member A2 | 4.55E-05 | 1.54855 |
| 208078_s_at | SIK1 | salt-inducible kinase 1 | 3.43E-05 | 1.55137 |
| 218858_at | DEPTOR | DEP domain containing MTOR-interacting protein | 7.35E-05 | 1.55505 |
| 202086_at | MX1 | myxovirus (influenza virus) resistance 1, interferon-inducible protein p78 (mouse) | 0.000646634 | 1.56692 |
| 206012_at | LEFTY1 /// LEFTY2 | left-right determination factor 1 /// left-right determination factor 2 | 0.000104455 | 1.57346 |
| 204698_at | ISG20 | interferon stimulated exonuclease gene 20kDa | 4.43E-05 | 1.57508 |
| 204655_at | CCL5 | chemokine (C-C motif) ligand 5 | 0.000154052 | 1.58564 |
| 227985_at | LOC100506098 | uncharacterized LOC100506098 | 0.000109807 | 1.60047 |
| 39248_at | AQP3 | aquaporin 3 (Gill blood group) | 4.15E-06 | 1.60088 |
| 207522_s_at | ATP2A3 | ATPase, Ca++ transporting, ubiquitous | 7.66E-05 | 1.60345 |
| 232687_at | GPRIN3 | GPRIN family member 3 | 6.53E-07 | 1.60885 |
| 210567_s_at | SKP2 | S-phase kinase-associated protein 2, E3 ubiquitin protein ligase | 1.25E-05 | 1.61428 |
| 209282_at | PRKD2 | protein kinase D2 | 9.68E-06 | 1.61919 |
| 201416_at | SOX4 | SRY (sex determining region Y)-box 4 | 2.94E-05 | 1.62277 |
| 229779_at | COL4A4 | collagen, type IV, alpha 4 | 0.000371304 | 1.62603 |
| 214162_at | ASXL3 | additional sex combs like transcriptional regulator 3 | 0.000459673 | 1.63623 |
| 201417_at | SOX4 | SRY (sex determining region Y)-box 4 | 2.94E-07 | 1.63777 |
| 209827_s_at | IL16 | interleukin 16 | 3.62E-05 | 1.63798 |
| 1555759_a_at | CCL5 | chemokine (C-C motif) ligand 5 | 9.76E-06 | 1.64719 |
| 1405_i_at | CCL5 | chemokine (C-C motif) ligand 5 | 2.64E-05 | 1.65555 |
| 1558920_at | SLC8A1-AS1 | SLC8A1 antisense RNA 1 | 0.00017087 | 1.65709 |
| 236008_at | RP11-305L7.3 | --- | 0.00011304 | 1.65888 |
| 208893_s_at | DUSP6 | dual specificity phosphatase 6 | 3.59E-05 | 1.67895 |
| 225810_at | MTMR10 | myotubularin related protein 10 | 4.68E-05 | 1.69354 |
| 228897_at | DERL3 | derlin 3 | 7.50E-05 | 1.70735 |
| 209846_s_at | BTN3A2 | butyrophilin, subfamily 3, member A2 | 6.44E-06 | 1.71321 |
| 213056_at | FRMD4B | FERM domain containing 4B | 9.46E-05 | 1.71347 |
| 233536_at | ASXL3 | additional sex combs like transcriptional regulator 3 | 0.00041384 | 1.71466 |
| 213888_s_at | TRAF3IP3 | TRAF3 interacting protein 3 | 5.29E-08 | 1.76453 |
| 208891_at | DUSP6 | dual specificity phosphatase 6 | 5.40E-07 | 1.85282 |
| 229721_x_at | DERL3 | derlin 3 | 0.000138673 | 1.85803 |
| 208892_s_at | DUSP6 | dual specificity phosphatase 6 | 6.73E-08 | 1.867 |
| 226961_at | PRR15 | proline rich 15 | 1.86E-05 | 1.86867 |
| 224772_at | NAV1 | neuron navigator 1 | 0.000163231 | 1.89347 |
| 235764_at | PRDM5 | PR domain containing 5 | 6.82E-06 | 1.92514 |
| 221911_at | ETV1 | ets variant 1 | 3.73E-05 | 2.37405 |

### Supplementary Table 6: Genes with a significant change in expression with EZH2 inhibition in the KMM1 cell line (p<0.05).

A negative fold changes indicates an increase in gene expression with EZH2 inhibition. ‘---‘ = probeset not recognised.

| Column ID | Gene Symbol | Gene Name | p-value (DMSO vs. EZH2 inhibitor) | Fold-Change (DMSO vs. EZH2 inhibitor) |
| --- | --- | --- | --- | --- |
| 1554766_s_at | --- | --- | 0.0011851 | -2.651 |
| 209395_at | CHI3L1 | chitinase 3-like 1 (cartilage glycoprotein-39) | 0.0151392 | -2.638 |
| 229450_at | IFIT3 | interferon-induced protein with tetratricopeptide repeats 3 | 0.0037082 | -2.379 |
| 209396_s_at | CHI3L1 | chitinase 3-like 1 (cartilage glycoprotein-39) | 0.01618 | -2.267 |
| 203767_s_at | STS | steroid sulfatase (microsomal), isozyme S | 0.0008492 | -2.218 |
| 203595_s_at | IFIT5 | interferon-induced protein with tetratricopeptide repeats 5 | 0.0026675 | -2.174 |
| 225167_at | FRMD4A | FERM domain containing 4A | 0.0067373 | -2.141 |
| 243198_at | TEX9 | testis expressed 9 | 1.47E-05 | -2.049 |
| 218918_at | MAN1C1 | mannosidase, alpha, class 1C, member 1 | 0.0111367 | -1.978 |
| 209921_at | SLC7A11 | solute carrier family 7 (anionic amino acid transporter light chain, xc- system), membe | 0.0412986 | -1.913 |
| 225168_at | FRMD4A | FERM domain containing 4A | 0.0061249 | -1.901 |
| 1558048_x_at | --- | --- | 0.0173926 | -1.859 |
| 217678_at | SLC7A11 | solute carrier family 7 (anionic amino acid transporter light chain, xc- system), member | 0.0377312 | -1.842 |
| 219412_at | RAB38 | RAB38, member RAS oncogene family | 0.0325322 | -1.812 |
| 217997_at | PHLDA1 | pleckstrin homology-like domain, family A, member 1 | 0.0491271 | -1.805 |
| 1553575_at | ND6 | NADH dehydrogenase, subunit 6 (complex I) | 0.0245755 | -1.776 |
| 213537_at | HLA-DPA1 | major histocompatibility complex, class II, DP alpha 1 | 0.0013312 | -1.771 |
| 1568609_s_at | LINC00623 /// LINC00869 /// LINC01138 /// LOC103091866 | long intergenic non-protein coding RNA 623 /// long intergenic non-protein coding RNA 8 | 0.0029326 | -1.771 |
| 211990_at | HLA-DPA1 | major histocompatibility complex, class II, DP alpha 1 | 0.0017953 | -1.755 |
| 206633_at | CHRNA1 | cholinergic receptor, nicotinic, alpha 1 (muscle) | 0.0021008 | -1.754 |
| 238900_at | HLA-DRB1 /// HLA-DRB3 /// HLA-DRB4 /// HLA-DRB5 /// LOC100996809 | major histocompatibility complex, class II, DR beta 1 /// major histocompatibility comp | 0.001475 | -1.749 |
| 207339_s_at | LTB | lymphotoxin beta (TNF superfamily, member 3) | 0.0041612 | -1.748 |
| 1560031_at | FRMD4A | FERM domain containing 4A | 0.0037234 | -1.741 |
| 224917_at | MIR21 /// VMP1 | microRNA 21 /// vacuole membrane protein 1 | 0.0003035 | -1.714 |
| 203769_s_at | STS | steroid sulfatase (microsomal), isozyme S | 0.0129977 | -1.71 |
| 229802_at | WISP1 | WNT1 inducible signaling pathway protein 1 | 0.0115753 | -1.71 |
| 208476_s_at | FRMD4A | FERM domain containing 4A | 0.006315 | -1.702 |
| 205943_at | TDO2 | tryptophan 2,3-dioxygenase | 0.000975 | -1.7 |
| 35974_at | LRMP | lymphoid-restricted membrane protein | 0.0065489 | -1.696 |
| 209906_at | C3AR1 | complement component 3a receptor 1 | 0.0037849 | -1.691 |
| 217967_s_at | FAM129A | family with sequence similarity 129, member A | 0.0217597 | -1.687 |
| 225606_at | BCL2L11 | BCL2-like 11 (apoptosis facilitator) | 7.48E-05 | -1.668 |
| 1558508_a_at | C1orf53 | chromosome 1 open reading frame 53 | 0.0031283 | -1.666 |
| 201137_s_at | HLA-DPB1 | major histocompatibility complex, class II, DP beta 1 | 0.0002701 | -1.66 |
| 205862_at | GREB1 | growth regulation by estrogen in breast cancer 1 | 0.0018723 | -1.658 |
| 211991_s_at | HLA-DPA1 | major histocompatibility complex, class II, DP alpha 1 | 0.0001243 | -1.657 |
| 203770_s_at | STS | steroid sulfatase (microsomal), isozyme S | 0.0003298 | -1.653 |
| 203768_s_at | STS | steroid sulfatase (microsomal), isozyme S | 0.002958 | -1.653 |
| 202660_at | ITPR2 | inositol 1,4,5-trisphosphate receptor, type 2 | 5.36E-05 | -1.651 |
| 226034_at | DUSP4 | dual specificity phosphatase 4 | 0.0015563 | -1.65 |
| 224451_x_at | ARHGAP9 | Rho GTPase activating protein 9 | 0.0034897 | -1.649 |
| 204897_at | PTGER4 | prostaglandin E receptor 4 (subtype EP4) | 0.0056511 | -1.644 |
| 217996_at | PHLDA1 | pleckstrin homology-like domain, family A, member 1 | 0.0356821 | -1.641 |
| 201212_at | LGMN | legumain | 0.0236064 | -1.641 |
| 239765_at | CPEB3 | cytoplasmic polyadenylation element binding protein 3 | 0.0013489 | -1.627 |
| 226189_at | ITGB8 | integrin, beta 8 | 0.0068652 | -1.624 |
| 220615_s_at | FAR2 | fatty acyl CoA reductase 2 | 0.0030327 | -1.624 |
| 227598_at | ZBED6CL | ZBED6 C-terminal like | 0.0012403 | -1.621 |
| 203932_at | HLA-DMB | major histocompatibility complex, class II, DM beta | 0.0009195 | -1.617 |
| 220595_at | PDZRN4 | PDZ domain containing ring finger 4 | 0.0169799 | -1.615 |
| 204014_at | DUSP4 | dual specificity phosphatase 4 | 0.0223051 | -1.611 |
| 235666_at | ITGA8 | integrin, alpha 8 | 0.000371 | -1.61 |
| 215111_s_at | TSC22D1 | TSC22 domain family, member 1 | 0.0005846 | -1.603 |
| 212077_at | CALD1 | caldesmon 1 | 0.0096155 | -1.598 |
| 233375_at | EFCAB2 | EF-hand calcium binding domain 2 | 0.0051077 | -1.596 |
| 221008_s_at | ETNPPL | ethanolamine-phosphate phospho-lyase | 0.0010448 | -1.595 |
| 211719_x_at | FN1 | fibronectin 1 | 0.0112085 | -1.59 |
| 239092_at | ITGA8 | integrin, alpha 8 | 0.0014242 | -1.59 |
| 205651_x_at | RAPGEF4 | Rap guanine nucleotide exchange factor (GEF) 4 | 0.0284394 | -1.588 |
| 222343_at | BCL2L11 | BCL2-like 11 (apoptosis facilitator) | 0.0004464 | -1.586 |
| 209480_at | HLA-DQB1 | major histocompatibility complex, class II, DQ beta 1 | 0.0014565 | -1.584 |
| 201341_at | ENC1 | ectodermal-neural cortex 1 (with BTB domain) | 0.0017822 | -1.575 |
| 204103_at | CCL4 | chemokine (C-C motif) ligand 4 | 0.0122567 | -1.574 |
| 202743_at | PIK3R3 | phosphoinositide-3-kinase, regulatory subunit 3 (gamma) | 0.0076343 | -1.569 |
| 212724_at | RND3 | Rho family GTPase 3 | 0.0055705 | -1.569 |
| 1558143_a_at | BCL2L11 | BCL2-like 11 (apoptosis facilitator) | 0.0001106 | -1.566 |
| 206345_s_at | PON1 | paraoxonase 1 | 0.0245335 | -1.563 |
| 206796_at | WISP1 | WNT1 inducible signaling pathway protein 1 | 0.0011794 | -1.556 |
| AFFX-M27830_M_at | --- | --- | 0.0098926 | -1.555 |
| 228150_at | SEC16B | SEC16 homolog B (S. cerevisiae) | 0.0099507 | -1.539 |
| 214265_at | ITGA8 | integrin, alpha 8 | 0.0036233 | -1.537 |
| 236163_at | LIX1 | Lix1 homolog (chicken) | 0.0081729 | -1.534 |
| 1554168_a_at | SH3KBP1 | SH3-domain kinase binding protein 1 | 0.0042753 | -1.532 |
| 1555756_a_at | CLEC7A | C-type lectin domain family 7, member A | 0.0206465 | -1.518 |
| 212998_x_at | HLA-DQB1 /// LOC101060835 | major histocompatibility complex, class II, DQ beta 1 /// HLA class II histocompatibili | 0.012804 | -1.516 |
| 237753_at | IL21R | interleukin 21 receptor | 0.0006087 | -1.515 |
| 208894_at | HLA-DRA | major histocompatibility complex, class II, DR alpha | 0.0065285 | -1.514 |
| 211600_at | PTPRO | protein tyrosine phosphatase, receptor type, O | 0.0103737 | -1.514 |
| 214696_at | MIR22 /// MIR22HG | microRNA 22 /// MIR22 host gene (non-protein coding) | 0.0032821 | -1.514 |
| 236191_at | --- | --- | 0.0032862 | -1.513 |
| 218113_at | TMEM2 | transmembrane protein 2 | 0.0018633 | -1.51 |
| 232277_at | SLC28A3 | solute carrier family 28 (concentrative nucleoside transporter), member 3 | 0.0052359 | -1.508 |
| 226885_at | RNF217 | ring finger protein 217 | 0.0036851 | -1.508 |
| 223082_at | SH3KBP1 | SH3-domain kinase binding protein 1 | 0.0028349 | -1.506 |
| 206834_at | HBD | hemoglobin, delta | 0.0097707 | -1.506 |
| 224783_at | UBALD2 | UBA-like domain containing 2 | 0.0189511 | 1.5035 |
| 206641_at | TNFRSF17 | tumor necrosis factor receptor superfamily, member 17 | 0.0006201 | 1.5079 |
| 1570253_a_at | RHEBL1 | Ras homolog enriched in brain like 1 | 0.0074737 | 1.5095 |
| 226303_at | PGM5 | phosphoglucomutase 5 | 0.0034413 | 1.5241 |
| 201925_s_at | CD55 | CD55 molecule, decay accelerating factor for complement (Cromer blood group) | 0.0019238 | 1.5409 |
| 230403_at | RFX3 | regulatory factor X, 3 (influences HLA class II expression) | 0.0054171 | 1.5436 |
| 238488_at | IPO11 /// LRRC70 | importin 11 /// leucine rich repeat containing 70 | 0.0029375 | 1.545 |
| 212642_s_at | HIVEP2 | human immunodeficiency virus type I enhancer binding protein 2 | 0.0218944 | 1.5452 |
| 216986_s_at | IRF4 | interferon regulatory factor 4 | 0.0226636 | 1.5624 |
| 210942_s_at | ST3GAL6 | ST3 beta-galactoside alpha-2,3-sialyltransferase 6 | 0.0067228 | 1.5719 |
| 235764_at | PRDM5 | PR domain containing 5 | 0.0033535 | 1.5769 |
| 230900_at | CCDC110 | coiled-coil domain containing 110 | 0.0060311 | 1.5809 |
| 212641_at | HIVEP2 | human immunodeficiency virus type I enhancer binding protein 2 | 0.0235656 | 1.6048 |
| 227787_s_at | MED30 | mediator complex subunit 30 | 0.0001597 | 1.6062 |
| 224785_at | UBALD2 | UBA-like domain containing 2 | 0.010802 | 1.6545 |
| 212698_s_at | SEPT10 | septin 10 | 0.0074959 | 1.6722 |
| 226018_at | MTURN | maturin, neural progenitor differentiation regulator homolog (Xenopus) | 0.0015597 | 1.688 |
| 206632_s_at | APOBEC3B | apolipoprotein B mRNA editing enzyme, catalytic polypeptide-like 3B | 0.047522 | 1.7039 |
| 221911_at | ETV1 | ets variant 1 | 0.0041575 | 1.7309 |
| 206121_at | AMPD1 | adenosine monophosphate deaminase 1 | 0.0006838 | 1.8012 |
| 36711_at | MAFF | v-maf avian musculoaponeurotic fibrosarcoma oncogene homolog F | 0.0395261 | 3.3128 |

## Supplementary Figures

### Supplementary Figure 1: Progression free survival (PFS) in the Myeloma IX and UAMS datasets

1. Kaplan-Meier curves showing PFS in the MyIX dataset (A) comparing high EZH2 mRNA (>8.24, log 2 expression value, n=31) to all others (n=228). Median PFS 1.09 years (95% CI [0.89, 1.77]) vs 1.5 (95% CI [1.24, 1.67]). Logrank P = 0.023 and (B) in UAMS dataset comparing High EZH2 (>9.32, log 2 expression value n=254) to all others (n=967). Median PFS 2.9 years (95% CI [2.29, 3.57]) vs 7.15 (95% CI [6.37, 7.66]). Logrank P = 2.8e-14


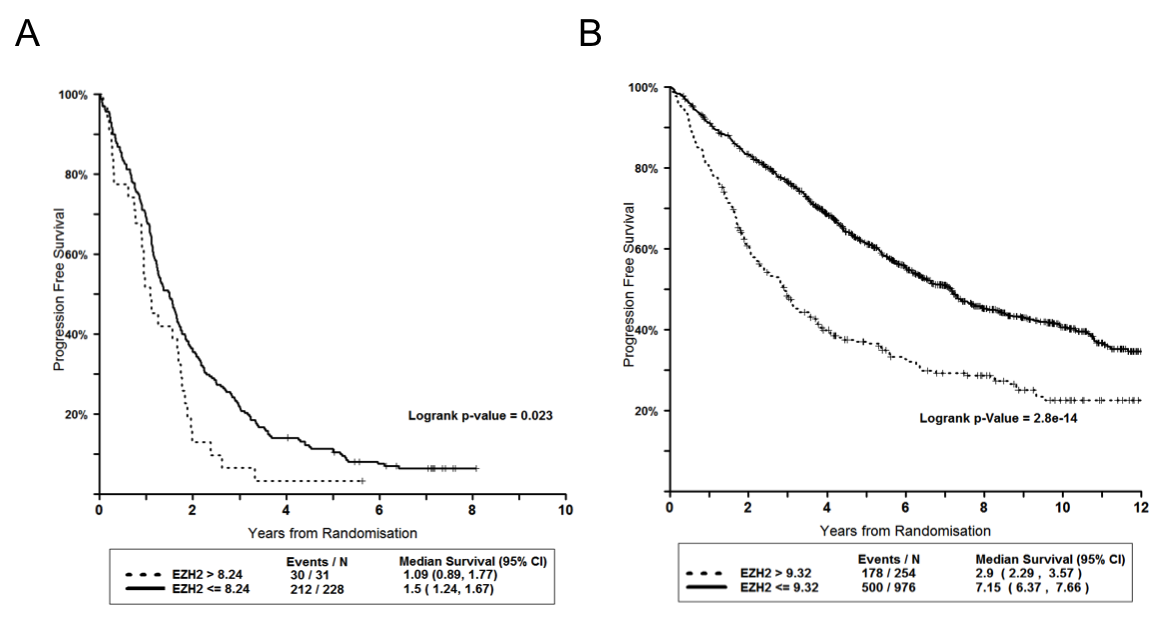


### Supplementary Figure 2: Further details of the viability analyses

A) EZH2, H3K27me3, H3K36me2 immunoblotting of lysates from untreated cell lines as indicated. Actin and total H3 were used as the loading control. Blots shown are representative of two independent experiments.

B) Cell viability determined using the WST1 assay (normalised to DMSO control) in a panel of 5 myeloma cell lines incubated with increasing concentrations of EZH2 inhibitor (EPZ005687) for 24 (left) and 48 (right) hours. The GI50 for each cell line (calculated using Graphpad Prism software) is shown. Graph shows mean and SEM of 2 independent biological replicates.

C) RPMI8226 cells were co-cultured with the bone marrow stromal cell line HS5 (GFP tagged) for 72 hours in the presence of the indicated concentration of EPZ005687 or vehicle control (DMSO). Cells were then stained with Annexin V and DAPI prior to flow cytometry analysis. Results show the % cell viability (of DMSO) measured as the % of cells that were Annexin V and PI negative within the GFP negative fraction. Mean and SEM of 3 independent experiments are shown. Unpaired t-tests were performed to compare the results at each concentration. Significant differences (p<0.05) are shown by *.

D) Proliferation of each cell line was assessed daily using the WST-1 assay. Colourimetric readouts after incubation at the indicated time points are expressed relative to baseline with the mean and SEM of 3 independent experiments shown.

E) Peripheral blood mononuclear cells were isolated from 3 healthy donors and incubated with EPZ005687 over 72 hours at the concentration indicated. Their viability was then assessed using the Annexin V / PI assay. Raw data and the mean value at each concentration are shown.

F) HS5-GFP cells (n=3) were incubated with EPZ005687 over 72 hours at the concentration indicated. Their viability was then assessed using the Annexin V / PI assay. The mean and SEM for viability of HS5-GFP cells at each concentration are shown.


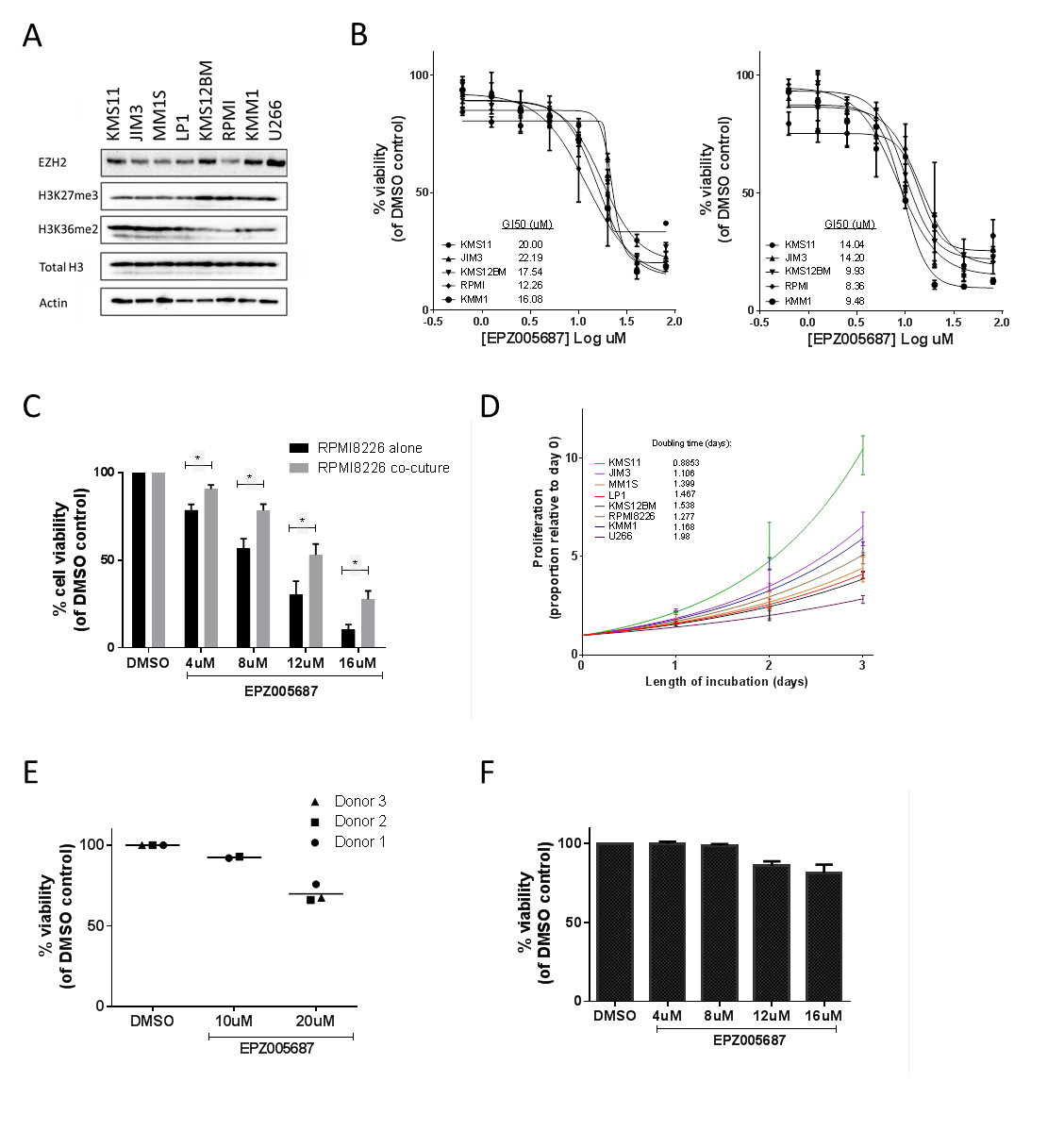


### Supplementary Figure 3: Confirmatory viability experiments with a second EZH2 inhibitor UNC1999 and its negative control compound UNC2400

A) Cell viability determined using the WST1 assay (normalised to DMSO control) in a panel of 8 myeloma cell lines incubated with increasing concentrations of EZH2 inhibitor (UNC1999) for 72 hours. The GI50 for each cell line (calculated using Graphpad Prism software) is shown. Graph shows mean and SEM of at least 3 independent biological replicates.

B) Cell viability determined using the WST1 assay (normalised to DMSO control) in (i) KMS11 and (ii) KMM1 cell lines incubated with increasing concentrations of EZH2 inhibitor (UNC1999) and its negative control compound (UNC2400) for 72 hours. Graph shows mean and SEM of 4 independent biological replicates.

C) Cell viability determined using the WST1 assay (normalised to DMSO control) was performed in parallel with either EPZ005687 or UNC1999 at increasing concentrations in KMS11, KMM1 and JIM3 cell lines for 6 days. Graph shows mean and SEM for at least 3 independent replicate experiments in each cell line. One sample t-tests were performed to look for a significant reduction in viability at 4uM compared to 100%. Those with p values <0.05 are indicated by *. Experiments using the concentration 1uM were not performed with EPZ005687.

D) Cell viability determined using the WST1 assay (normalised to DMSO control) was performed in parallel with UNC1999 and its negative control UNC2400 at increasing concentratons in KMS11, KMM1 and JIM3 cell lines for 6 days. Graph shown mean and SEM for at least 2 independent replicate experiments in each cell line. Unpaired t-tests were performed to compare the results at each concentration for each cell line. Significant differences (p<0.05) are shown by *.


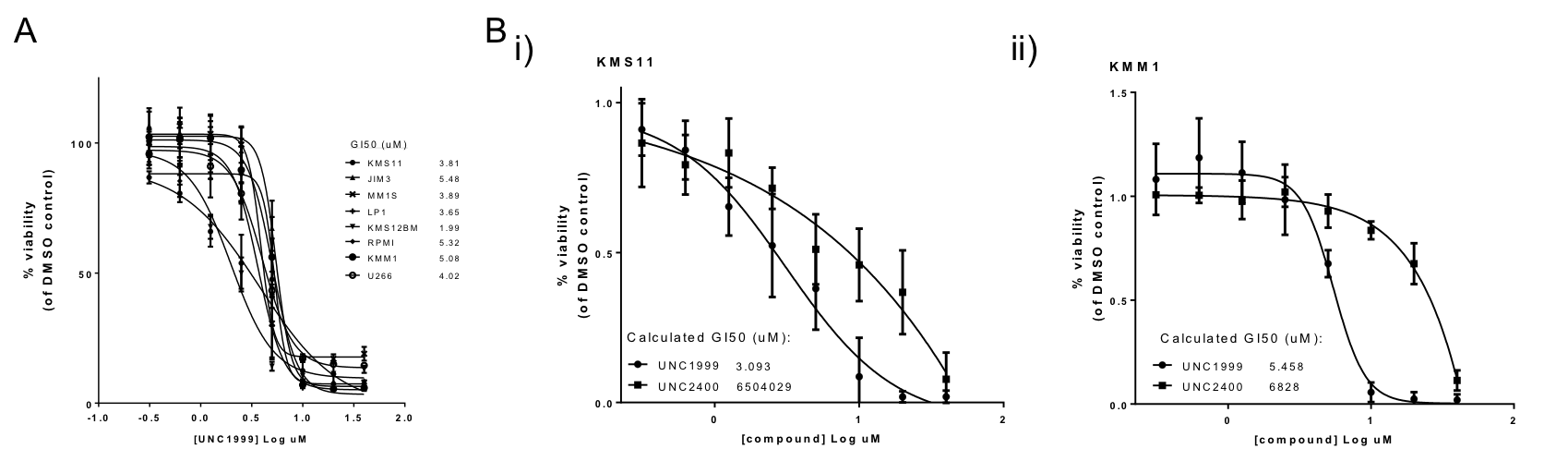


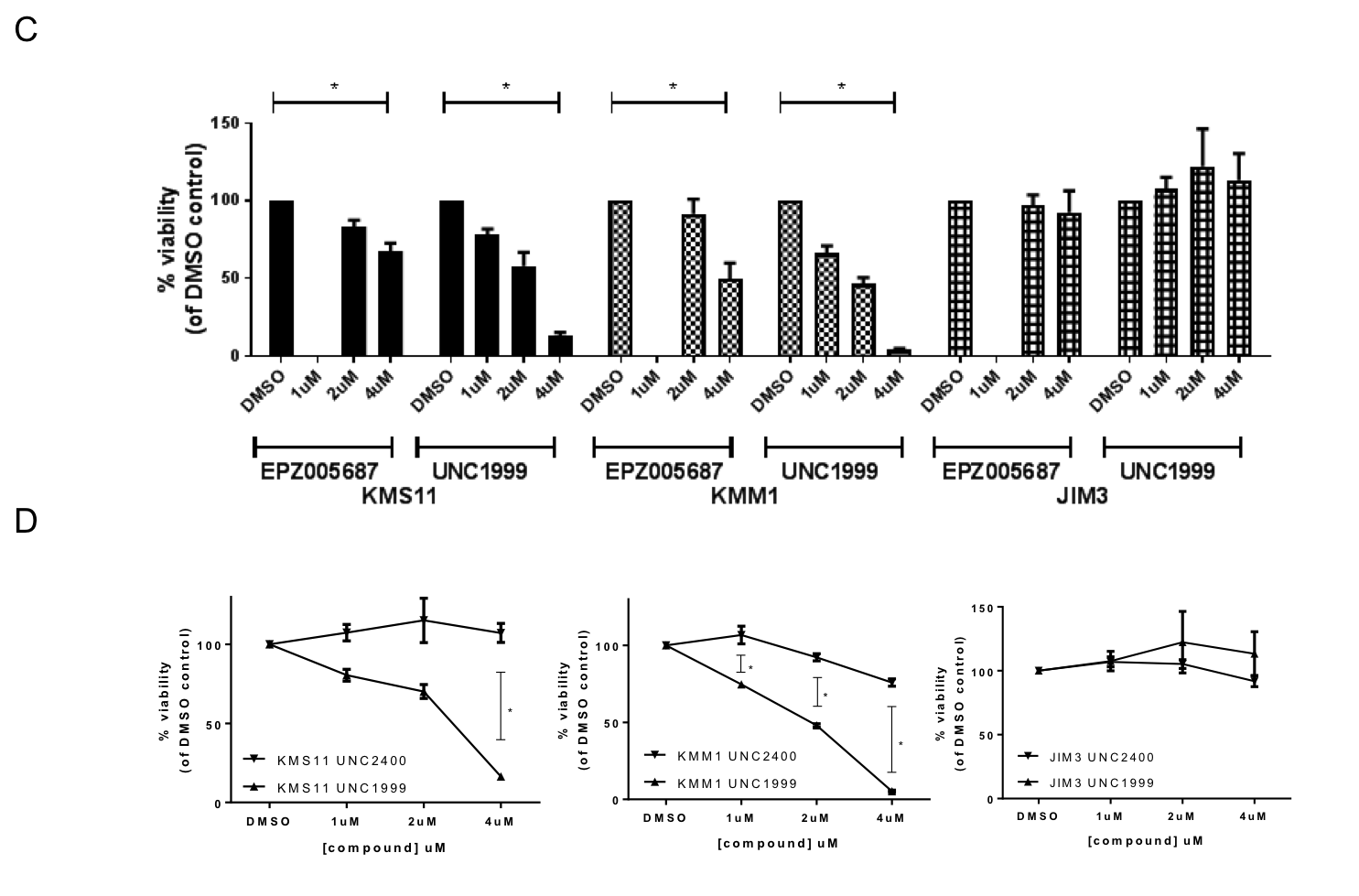


### Supplementary Figure 4: Confirmatory apoptosis experiments

A) PI staining and measuring the percentage of cells in the sub-G1 phase in KMS11 and KMM1 cell lines. A one way ANOVA followed by multiple comparisons to DMSO control was performed.

B) CaspaseGlo assay measuring caspase 3/7 activity measured by luminescence in KMS11 and KMM1 cell lines. One sample t-tests were performed to look for a significant increase at each concentration compared to 1.

C) Immunoblotting for PARP cleavage using whole cell lysates of KMS11 and KMM1 cell lines.

All graphs show the mean and SEM of at least 3 independent replicates. Blots shown are representative of two independent experiments. Statistical significance (p<0.05) is indicated by *.


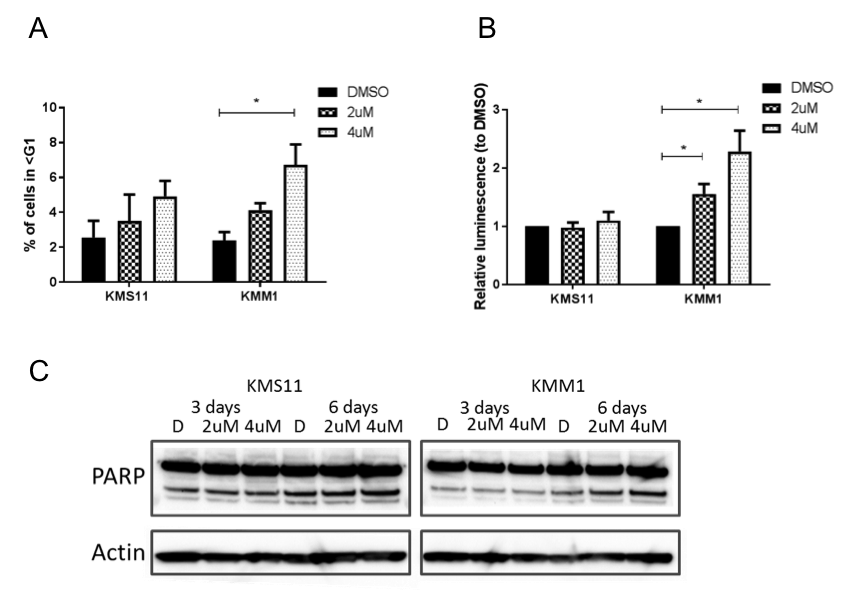


### Supplementary Figure 5: qRT-PCR experiment with the negative control compounds UNC2400

Fold change in mRNA levels in UNC2400 treated KMS11 and KMM1 cell lines at 3 and 6 days, compared to DMSO control at the same time point, measured by qRT-PCR. Graphs show mean and SEM for 2 independent replicate experiments. GAPDH was used as the internal control.


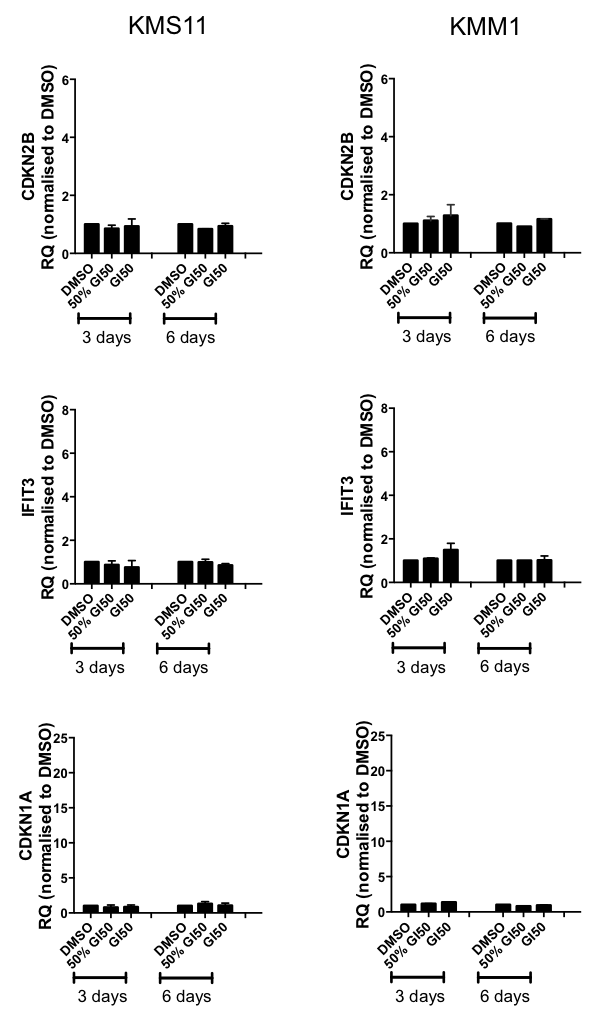


### Supplementary Figure 6: Correlation between EZH2 and CDKN1A expression.

High density scatter plot demonstrating the correlation between EZH2 mRNA expression and CDKN1A mRNA expression in the UAMS patient dataset. R= -0.170, p<0.0001.


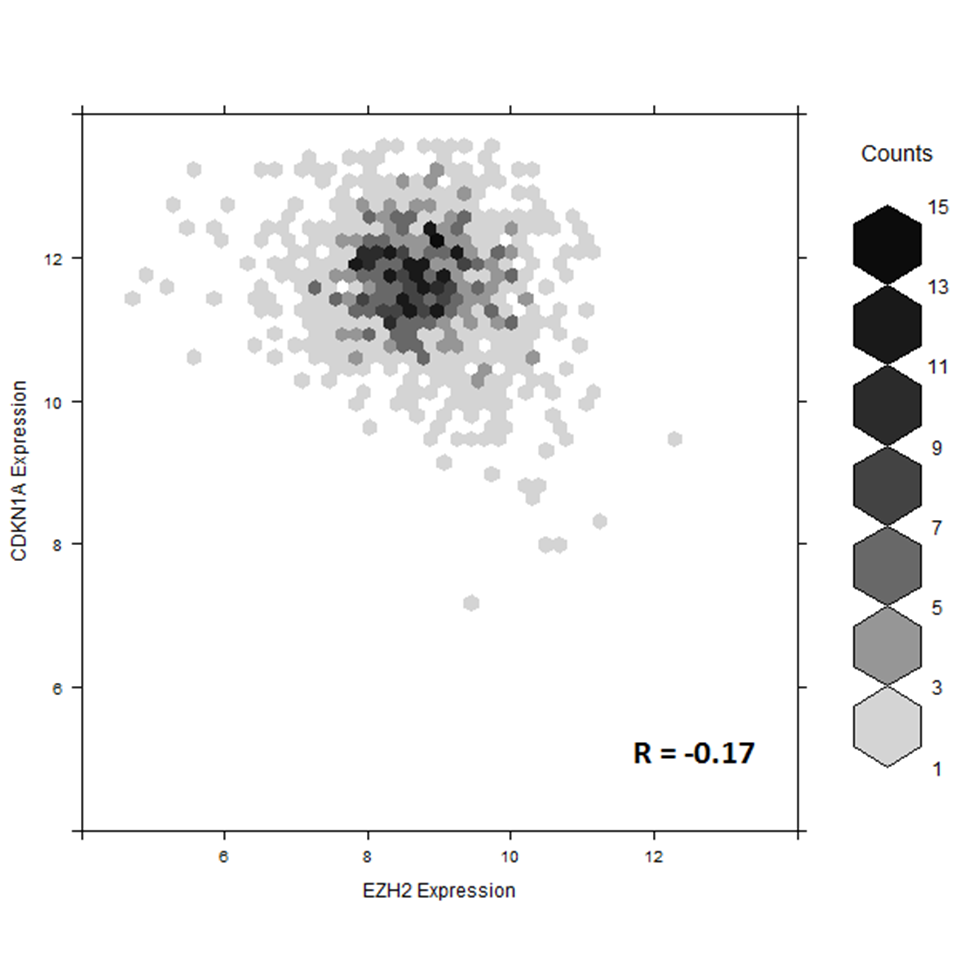


### Supplementary Figure 7: Further analysis of H3K27 methylation changes in response to EZH2 inhibition

A) H3K27me3 immunoblotting of lysates from KMS11 and KMM1 cell lines incubated with UNC199 9 over 3 or 6 days as indicates. Actin and total H3 were used as the loading control. Blots shown are representative of two independent experiments.

B) H3K27me3, H3K27me2, H3K27me1, H3K36m2 and EZH2 immunoblotting in a panel of 8 myeloma cell lines. Total H3 was used as a loading control. Blots shown are representative of two independent experiments.


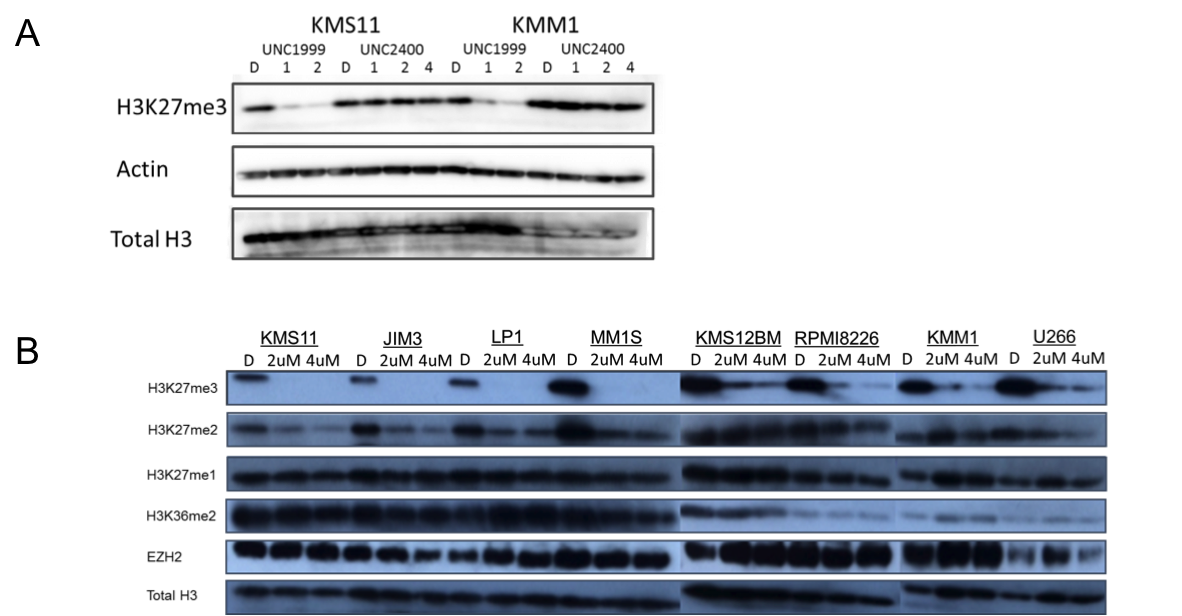


### Supplementary Figure 8: Further analysis of H3K27 methylation changes in response to EZH2 inhibition

Fold change in mRNA levels, compared to DMSO control, in a panel of 8 myeloma cell lines incubated with EPZ005687 for 6 days, measured by qRT-PCR. Graphs show mean and SEM for 3 independent replicate experiments for the genes indicated. GAPDH was used as the internal control.


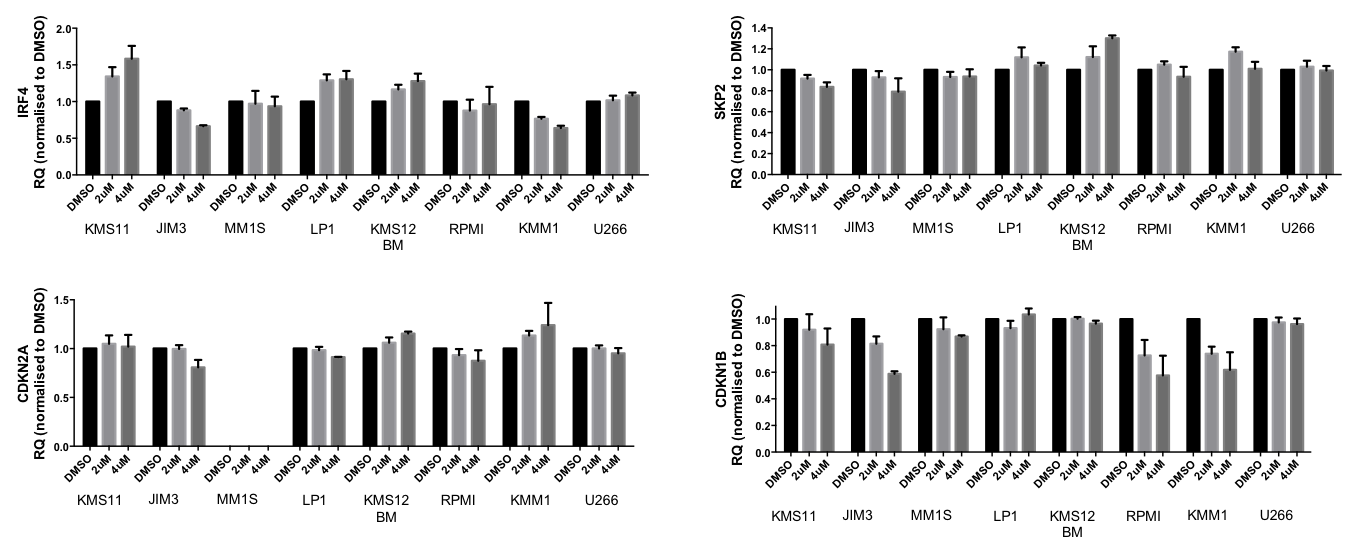


## Supplementary References:

1. Morgan, G.J., et al., *Effects of induction and maintenance plus long-term bisphosphonates on bone disease in patients with multiple myeloma: the Medical Research Council Myeloma IX Trial.* Blood, 2012. **119**(23): p. 5374-83.

2. Morgan, G.J., et al., *Cyclophosphamide, thalidomide, and dexamethasone (CTD) as initial therapy for patients with multiple myeloma unsuitable for autologous transplantation.* Blood, 2011. **118**(5): p. 1231-8.

3. Morgan, G.J., et al., *Cyclophosphamide, thalidomide, and dexamethasone as induction therapy for newly diagnosed multiple myeloma patients destined for autologous stem-cell transplantation: MRC Myeloma IX randomized trial results.* Haematologica, 2012. **97**(3): p. 442-50.

4. Morgan, G.J., et al., *Long-Term Follow-Up of MRC Myeloma IX Trial: Survival Outcomes with Bisphosphonate and Thalidomide Treatment.* Clin Cancer Res, 2013.

5. Morgan, G.J., et al., *Effects of zoledronic acid versus clodronic acid on skeletal morbidity in patients with newly diagnosed multiple myeloma (MRC Myeloma IX): secondary outcomes from a randomised controlled trial.* Lancet Oncol, 2011. **12**(8): p. 743-52.

6. Barlogie, B., et al., *Curing myeloma at last: defining criteria and providing the evidence.* Blood, 2014. **124**(20): p. 3043-51.

7. Barlogie, B., et al., *Superiority of tandem autologous transplantation over standard therapy for previously untreated multiple myeloma.* Blood, 1997. **89**(3): p. 789-93.

8. Barlogie, B., et al., *Thalidomide arm of Total Therapy 2 improves complete remission duration and survival in myeloma patients with metaphase cytogenetic abnormalities.* Blood, 2008. **112**(8): p. 3115-21.

9. Nair, B., et al., *Superior results of Total Therapy 3 (2003-33) in gene expression profiling-defined low-risk multiple myeloma confirmed in subsequent trial 2006-66 with VRD maintenance.* Blood, 2010. **115**(21): p. 4168-73.

10. van Rhee, F., et al., *Total Therapy 3 for multiple myeloma: prognostic implications of cumulative dosing and premature discontinuation of VTD maintenance components, bortezomib, thalidomide, and dexamethasone, relevant to all phases of therapy.* Blood, 2010. **116**(8): p. 1220-7.

11. Zangari, M., et al., *Eight-year median survival in multiple myeloma after total therapy 2: roles of thalidomide and consolidation chemotherapy in the context of total therapy 1.* Br J Haematol, 2008. **141**(4): p. 433-44.

12. Jethava, Y., et al., *Dose-dense and less dose-intense Total Therapy 5 for gene expression profiling-defined high-risk multiple myeloma.* Blood Cancer J, 2016. **6**(7): p. e453.

13. Zhan, F., et al., *The molecular classification of multiple myeloma.* Blood, 2006. **108**(6): p. 2020-8.

14. van Haaften, G., et al., *Somatic mutations of the histone H3K27 demethylase gene UTX in human cancer.* Nat Genet, 2009. **41**(5): p. 521-3.
